# Geographic and host distribution of haemosporidian parasite lineages from birds of the family Turdidae

**DOI:** 10.1186/s12936-020-03408-0

**Published:** 2020-09-15

**Authors:** Josef Harl, Tanja Himmel, Gediminas Valkiūnas, Mikas Ilgūnas, Támas Bakonyi, Herbert Weissenböck

**Affiliations:** 1 Institute of Pathology, Department for Pathobiology, University of Veterinary Medicine Vienna, Veterinaerplatz 1, 1210 Vienna, Austria; 2 Nature Research Centre, Akademijos 2, 08412 Vilnius, Lithuania; 3 Institute of Virology, Department for Pathobiology, University of Veterinary Medicine Vienna, Veterinaerplatz 1, 1210 Vienna, Austria

**Keywords:** *CytB*, Haplotype networks, GenBank, MalAvi database, *Plasmodium*, *Haemoproteus*, *Leucocytozoon*

## Abstract

**Background:**

Haemosporidians (Apicomplexa, Protista) are obligate heteroxenous parasites of vertebrates and blood-sucking dipteran insects. Avian haemosporidians comprise more than 250 species traditionally classified into four genera, *Plasmodium*, *Haemoproteus*, *Leucocytozoon*, and *Fallisia*. However, analyses of the mitochondrial *CytB* gene revealed a vast variety of lineages not yet linked to morphospecies. This study aimed to analyse and discuss the data of haemosporidian lineages isolated from birds of the family Turdidae, to visualise host and geographic distribution using DNA haplotype networks and to suggest directions for taxonomy research on parasite species.

**Methods:**

Haemosporidian *CytB* sequence data from 350 thrushes were analysed for the present study and complemented with *CytB* data of avian haemosporidians gathered from Genbank and MalAvi database. Maximum Likelihood trees were calculated to identify clades featuring lineages isolated from Turdidae species. For each clade, DNA haplotype networks were calculated and provided with information on host and geographic distribution.

**Results:**

In species of the Turdidae, this study identified 82 *Plasmodium*, 37 *Haemoproteus*, and 119 *Leucocytozoon* lineages, 68, 28, and 112 of which are mainly found in this host group. Most of these lineages cluster in the clades, which are shown as DNA haplotype networks. The lineages of the *Leucocytozoon* clades were almost exclusively isolated from thrushes and usually were restricted to one host genus, whereas the *Plasmodium* and *Haemoproteus* networks featured multiple lineages also recovered from other passeriform and non-passeriform birds.

**Conclusion:**

This study represents the first attempt to summarise information on the haemosporidian parasite lineages of a whole bird family. The analyses allowed the identification of numerous groups of related lineages, which have not been linked to morphologically defined species yet, and they revealed several cases in which *CytB* lineages were probably assigned to the wrong morphospecies. These taxonomic issues are addressed by comparing distributional patterns of the *CytB* lineages with data from the original species descriptions and further literature. The authors also discuss the availability of sequence data and emphasise that MalAvi database should be considered an extremely valuable addition to GenBank, but not a replacement.

## Background

Haemosporidians (Haemosporida, Apicomplexa) represent one of the most diverse and ubiquitous groups of protozoan parasites. More than 250 species in four genera were described from bird hosts based on the data in [1] and more recent species descriptions. The genus *Haemoproteus* includes more than 170 species classified into two subgenera, with over 160 *Parahaemoproteus* and less than ten *Haemoproteus* species. The genus *Plasmodium* comprises at least 55 avian malaria species in five subgenera [2]. The genus *Leucocytozoon* also comprises about 50 species, including a single species of the subgenus *Akiba*, and the genus *Fallisia* includes a single avian parasite species in the subgenus *Plasmodioides*. However, molecular genetic data indicate that these numbers are provisional due to current active taxonomical work, and they likely represent only the tip of the iceberg of true species diversity in avian haemosporidians.

Most DNA-barcoding approaches of multicellular eukaryotes target sections of mitochondrial (mt) genes, particularly of the *Cytochrome c oxidase subunit I* (*COI*) and the *Cytochrome b* (*CytB*). The *CytB* became the reference gene for DNA-barcoding approaches of both avian haemosporidians and birds. These mitochondrial genes are particularly useful because they are ubiquitous in eukaryotes and extremely conserved in length, allowing the alignment and comparison of sequences between both closely and distantly related taxa. Moreover, mitochondrial genomes are haploid, featuring genes in single copies. The first PCR protocols for targeting a wider range of avian haemosporidian parasites were developed by [3] and then refined by [4, 5]. These PCR approaches allow amplification and sequencing of a 478 base pair (bp) fragment of the *CytB* of *Haemoproteus* and *Plasmodium* species as well as a 476 bp fragment of *Leucocytozoon* species, and they are the most widely used primer sets in avian haemosporidian research. This *CytB* section became accepted as the common DNA-barcode region of avian haemosporidians. Alternative PCR protocols covering the same sequence region were published in several other studies [e.g., 6–9]. As a consequence of the vastly increasing number of *CytB* sequences, [10] established a database aiming to assign names to all unique lineages and to summarise data on their geographic and host distribution. The so-called MalAvi database (http://130.235.244.92/Malavi/) currently features about 3500 unique lineages covering the complete (or almost complete) DNA-barcode region, and almost 12,000 individual records summarizing data of 30,000 parasite samples. The database became essential in unifying the recognition and naming of new haemosporidian lineages, and it vastly promoted studies linking morphologically described species to certain *CytB* lineages. Since its foundation in 2009, the MalAvi database was used in the majority of molecular genetic studies on avian haemosporidians, having been cited in over 500 publications. Moreover, the database complements the data of numerous publications for which data on individual samples were neither submitted to NCBI GenBank nor provided as supporting information.

### Birds of the family Turdidae

Species of the Turdidae Rafinesque, 1815, or thrushes, are small to medium-sized songbirds of the order Passeriformes, which inhabit all biogeographic realms. Most thrushes forage on the ground, where they feed on insects, earthworms, land snails, and fruit. Migration behavior can vary substantially between species, ranging from long-distance migrants to resident birds. The family currently includes about 160 species classified into 20 genera [11]. The most species-rich genera are *Turdus* (80 species), *Geokichla* (18), *Zoothera* (15), *Catharus* (12), *Myadestes* (12), *Cochoa* (4), and *Sialia* (3), while the other genera include only one or two species each. Of these genera, four are mentioned here because data were also included in the present study: *Entomodestes* (2), *Neocossyphus* (2), *Hylocichla* (1), and *Ixoreus* (1) [11]. Previously, birds of the family Muscicapidae were also classified as Turdidae, but they do not form a monophyletic group with them and are now considered an independent group [12].

### Haemosporidian parasites of birds of the family Turdidae

So far, 14 haemosporidian parasite species have been described from bird hosts of the family Turdidae. These include five *Plasmodium*, four *Haemoproteus*, and four *Leucocytozoon* species (Table 1). One parasite species, *Plasmodium lutzi* was described from the Grey-necked wood rail Aramides cajaneus (Gruiformes) in Brazil, but is considered to be common in thrushes [13].

**Table 1:** Avian haemosporidian parasite species described from Turdidae hosts

Parasite species	Authors	Type host species	Type locality
*P.* (*Giovannolaia*) *circumflexum*	Kikuth [94]	*Turdus pilaris*	Germany
*P.* (*Haemamoeba*) *giovannolai*	Corradetti et al. [84]	*Turdus merula*	Lazio (Italy)
*P.* (*Haemamoeba*) *lutzi*	Lucena [88]	*Aramides cajaneus* (Gruiformes)	São Paulo (Brazil)
*P.* (*Haemamoeba*) *matutinum*	Huff [76]	*Turdus migratorius*	Illinois (USA)
*P.* (*Novyella*) *hexamerium*	Huff [68]	*Sialia sialis*	Illinois (USA)
*P.* (*Novyella*) *vaughani*	Novy and MacNeal [55]	*Turdus migratorius*	Michigan (USA)
*H.* (*Parahaemoproteus*) *fallisi*	Bennett and Campbell [99]	*Turdus migratorius*	Newfoundland (Canada)
*H.* (*Parahaemoproteus*) *geocichlae*	Cleland and Johnston [101]	*Zoothera lunulata*	New South Wales (Australia)
*H.* (*Parahaemoproteus*) *homominutus*	Valkiūnas et al. [46]	*Turdus viscivorus*	Lithuania
*H.* (*Parahaemoproteus*) *minutus*	Valkiūnas and Iezhova [102]	*Turdus merula*	Lithuania
*L.* (*Leucocytozoon*) *dubreuili*	Mathis and Léger [113]	*Turdus* sp. (‘grive’)	Tonkin (Vietnam)
*L.* (*Leucocytozoon*) *giovannolai* [probably synonym of *L. dubreuili*]	Travassos Santos Dias [116]	*Turdus iliacus*	Italy
*L.* (*Leucocytozoon*) *maccluri*	Greiner [118]	*Zoothera marginata*	Chiang Mai (Thailand)
*L.* (*Leucocytozoon*) *mirandae*	França [117]	*Turdus merula*	Portugal

### Sequence data published from Turdidae hosts

Thrushes were sampled for various studies on avian haemosporidians, but none of the molecular genetic studies particularly dealt with haemosporidian parasites of this host family. Most of the haemosporidian *CytB* sequences of thrushes come from a few ecological studies screening samples of large numbers of passeriform birds. [8] analysed *CytB* sequences recovered from more than 2300 birds from Western Europe, Western Russia, Western Asia, and Northern Africa, 186 of which originated from thrushes. [14] studied 69 bird communities from all over the Americas and published at least 86 sequences isolated from thrushes. Further data on haemosporidian parasites of Alaskan birds were published by [15, 16], featuring more than 100 sequences isolated from thrushes. Data from American thrushes were also published in ecological studies of [17–19]. More data from haemosporidians of American thrushes were published in [14, 20–25]. Additional sequence data originate from numerous other studies. All references for the *CytB* sequences used in this study are provided in Additional file 1.

### Geographic and host distribution of avian haemosporidian lineages from thrushes

Birds of the family Turdidae are among the most sampled host groups of avian groups, in which both the morphological and molecular diversity of haemosporidian parasites have been relatively well characterised in different zoogeographical regions [1, 26, 27]. This provided opportunities for the relatively representative comparative parasite taxonomic and distribution analysis and helped to determine unrecognised patterns in the distribution of avian haemosporidians. Among thrushes, *Turdus merula* is of particular interest because it was also introduced to European settlements in Australia and New Zealand together with its haemosporidian parasites, which potentially represent a threat to native bird species [28].

This study aimed at analyzing the geographic and host distribution of haemosporidian parasite lineages in thrushes worldwide. Records of haemosporidian parasite lineages were gathered from NCBI GenBank, MalAvi database, and related publications, and major clades containing multiple lineages common in thrushes were identified by performing phylogenetic analyses. To display the geographic and host distribution of the lineages contained within most of these clades, DNA haplotype networks were calculated. This method is particularly useful in population genetics to show the genetic diversity within species or to compare sequences of closely related species. Based on the geographic and host distribution as well as the similarity of lineages, groups of similar lineages potentially belonging to distinct parasite species were defined. Most of the lineages and groups of lineages identified have not been linked to morphospecies yet, and there are several cases in which the assignment was probably incorrect. To address these issues, the authors of the present study thoroughly discuss the information available on morphologically described haemosporidian parasites of thrushes and compare it with the distributional patterns of the *CytB* lineages found in this host group. The information summarised in this study provides directions for future taxonomic work on avian malaria and related haemosporidians on parasites species levels. The data may also help to understand the relationships between hosts and vectors and identify potential transmission areas.

## Materials and methods

### Sample preparation and PCR screening of Austrian thrushes

In the years 2003 to 2018, samples were collected from 310 individuals of *T. merula*, 36 individuals of *T. philomelos*, and two individuals of *T. pilaris*. Most samples (288) were taken from dead birds during a monitoring study at the Institute of Pathology (University of Veterinary Medicine Vienna) from 2003 to 2005. Another 15 samples were collected from dead birds between 2014 and 2017. After dissection, various organs were embedded in paraffin blocks and stored in the archive of the Institute of Pathology, whereas only native brain tissue was frozen and stored at − 80 °C for DNA isolation. Additional 43 blood samples were collected from living blackbirds and song thrushes received for treatment at the Bird and Reptile Clinic (Department for Companion Animals and Horses, University of Veterinary Medicine Vienna). Blood samples were taken by puncturing the brachial vein and using heparinised microcapillaries to transfer blood drops to high-grade filter papers Whatman™ 903 (GE Healthcare, Buckinghamshire, GB) of which DNA was isolated later.

From the tissue of dead birds, both DNA and RNA were isolated because these samples were originally used for the Usutu virus screenings [29–32]. Nucleic acids were extracted from 140 µl of homogenised brain tissue with the QIAamp Viral RNA Mini Kit (QIAGEN, Hilden, Germany) as described in [32]. From blood spots, DNA was extracted using the DNeasy Blood & Tissue Kit (QIAGEN, Venlo, Netherlands) following the manufacturer’s protocol for isolation of DNA from tissue. In the last step, two eluates of 100 µl were made from the same column, at 8000 rpm and 13,000 rpm, the second of which was used for the PCR screenings.

All 348 samples were screened for the presence of avian haemosporidians using the nested PCR-protocol established by [4], which allows obtaining mt *CytB* fragments of 476 bp for *Leucocytozoon* species and 478 bp for *Plasmodium* spp. and *Haemoproteus* spp. In the “nest 1” PCR, the primers HaemNFI (5′-CATATATTAAGAGAANTATGGAG-3′) and HaemNR3 (5′-ATAGAAAGATAAGAAATACCATTC-3′) were used. In the “nest 2” PCRs, the primers HaemF (5′-ATGGTGCTTTCGATATATGCATG-3′) and HaemR2 (5′-GCATTATCTGGATGTGATAATGGT-3′) were used to amplify the *CytB* of *Plasmodium* spp. and *Haemoproteus* spp., and HaemFL (5′-ATGGTGTTTTAGATACTTACATT-3′) and HaemR2L (5′-CATTATCTGGATGAGATAATGGIGC-3′) were used to amplify the *CytB* of *Leucocytozoon* spp. The PCRs were performed using the GoTaq^®^ G2 Flexi DNA Polymerase (Promega, Wisconsin, Madison, USA). The PCRs started with an initial denaturation for 2 min at 94 °C, followed by 35 cycles with 30 s at 94 °C, 30 s at 50 °C, 1 min at 72 °C, and a final extension for 10 min at 72 °C. Each 1 µl of “nest 1” PCR-product was used as a template in the “nest 2” PCRs.

The PCR products were sent to Microsynth Austria GmbH (Vienna, Austria) for purification and sequencing in both directions using the “nest 2” PCR primers. Raw forward and reverse sequences were manually aligned and electropherograms were checked in Bioedit v.7.0.8.0 [33]. Subsequently, all sequences were aligned and sorted with MAFFT v.7 [34] applying the default settings. If the sequences contained ambiguous characters indicating double infections, the electropherograms of the forward and reverse sequences were carefully rechecked and un-phased using DnaSP v.6.12.3 [35]. This method allows for the identification of lineages in mixed infections if all lineages in the data set are also present in single infections. It requires high-quality electropherograms allowing a clear assignment of double peaks and therefore should be used with caution. In multiple cases, the samples were also screened with the primers CytB_HPL_intF1 (5′-GAGAATTATGGAGTGGATGGTG-3′) and CytB_HPL_intR1 (5′-ATGTTTGCTTGGGAGCTGTAATC-3′) following the protocol by [36], which allows sequencing an 886 bp section of the *CytB* of all avian haemosporidians. The lineage names of the haplotypes were identified by performing BLAST searches in the MalAvi database (http://130.235.244.92/Malavi/, [10]). New haplotypes identified in the present study were assigned new lineage names and uploaded to the MalAvi database. Haemosporidian sequences isolated from all individual bird samples were uploaded to GenBank.

### CytB haplotype networks with haemosporidian parasite lineages of Turdidae birds

#### Data collection

(1) Multiple BLAST searches were performed in GenBank to gather *CytB* sequences of all haemosporidian parasites. Information on host species, localities, parasite species, and other data were extracted from the GenBank files and transferred to Microsoft Excel (Microsoft Office 365). (2) The *CytB* sequences were then aligned with MAFFT v.7 [34] applying the default settings. (3) The lengths of the sequences, the presence of ambiguity characters, and the overall sequence quality were determined using Bioedit v.7.0.8.0 [33] and Microsoft Excel. (4) In the next step, all sequences covering the complete (or almost complete) *CytB* DNA-barcode region were isolated, and those containing ambiguities or obvious sequencing errors (e.g. insertions, deletions, and faulty end-parts) were excluded from the data set. All records originating from non-avian hosts were removed as well, except for those coming from dipteran blood-sucking insects, which are potential vectors of the corresponding avian haemosporidian lineages. (5) The haemosporidian *CytB* sequences isolated from the Austrian thrushes screened for the present study were added to the data set and all sequences were realigned and sorted with MAFFT v.7. This alignment contained a total of 7902 avian haemosporidian *CytB* sequences. (6) The alignment was then divided into three partitions containing data of *Plasmodium*, *Haemoproteus*, and *Leucocytozoon* species with 2767, 2568, and 2567 sequences, respectively. (7) Maximum Likelihood (ML) trees were calculated for all three alignments using the W-IQ-TREE web server (http://iqtree.cibiv.univie.ac.at/; [37]), by applying the model GTR + G + I and performing 1.000 bootstrap replicates each. (8) Based on the ML trees and data from the GenBank files, all lineages originating from thrushes and clades featuring multiple Turdidae-specific lineages were identified. All of these clades obtained ML bootstrap support (bs) values of ≥ 90 and were considered “reciprocally monophyletic”. (9) For each of these lineages and clades, additional data were gathered from the corresponding publications, supporting information, and the MalAvi “Host and Sites” table. Only data from studies were included for which at least one sequence per lineage was submitted to GenBank because this allowed determining the lengths of the sequences and their quality.

#### DNA haplotype networks

For each of the clades containing multiple haemosporidian lineages isolated from thrushes, Median-Joining haplotype networks were calculated to visualise the geographic and host distribution of the lineages. The lineages contained in the networks each belong to clades as identified in the ML analysis. (1) The alignments for each clade were trimmed to 474 bp by removing the first two and last two (in case of the *Plasmodium* and *Haemoproteus* spp. sequences) nucleotides of the *CytB* DNA-barcode sequence. (2) Median-Joining haplotype networks were calculated with Network 5.0.1.0 (Fluxus Technology Ltd, Suffolk, England) applying the default settings. (3) The networks were graphically prepared and provided with information on host species and geographic region in Network Publisher v.2.1.2.3 (Fluxus Technology Ltd) and finalised with Adobe Illustrator CC v.2015 (Adobe Inc., San José, California, USA). For each network, two visual representations were prepared. The first displays the host distribution of lineages, whereby species of the family Turdidae were colour-coded. Only bird genera which revealed to be monophyletic in recent phylogenetic studies [e.g., 12] were considered as belonging to the Turdidae. Non-Turdidae birds are summarised as “Passeriformes others” and “non-Passeriformes” in most cases. The second visual representation shows the geographic distribution of lineages, whereby countries were assigned to geographic regions according to the United Nations geo-scheme with two slight modifications: the European regions Western-, Northern-, and Southern Europe were summarised as “Western Europe”, and Mexico was assigned to North America but not to Central America. Detailed information on host species, countries of origin, and related publications are provided in Additional file 1. For most clades, haplotype groups were defined based on the genetic similarity of the lineages, literature data, and the geographic and host distribution. Generally, in the *Plasmodium* and *Leucocytozoon* networks, lineages were considered belonging to one group if they differed by one to three bp from the central haplotype, and haplotype groups were considered to be distinct if they differed by five or more bp from each other. The haplotype groups in the network featuring lineages of *H. minutus* and closely related species partly differed in one few bp and therefore were defined based on literature data only.

#### Phylogenetic trees

To provide an overview of the diversity of *CytB* lineages, Maximum Likelihood (ML) and Bayesian Inference (BI) trees were calculated based on an alignment including all haemosporidian *CytB* lineages isolated from birds of the Turdidae (and related lineages from other birds included in the networks). A ML bootstrap consensus tree (1000 replicates) was calculated using the W-IQ-TREE web server (http://iqtree.cibiv.univie.ac.at/; [37]) and applying the model GTR + G + I, which was suggested as best fit for the data set in the model test according to the Bayesian inference criterion (BIC). The BI tree was calculated using MrBayes v.3.2.2 [38]. Applying the model GTR + G + I, the analysis was run for 5^10^ generations (2 runs with 4 chains, one of which was heated), sampling every thousandth tree. The first 25% of trees were discarded as burn-in, and a majority rule consensus tree was calculated based on the remaining 3.750 trees. Information on the geographic distribution of all lineages according to the United Nations geo-scheme were obtained from the MalAvi database.

## Results

### Haemosporidian parasites of *Turdus* species in Austria

Of 348 samples of *Turdus* spp. screened for the present study, 218 (prevalence 62.6%) were positive, whereby 177 featured mono-infections, 35 double infections, and 6 triple infections.

Of the 310 *T. merula* individuals, 182 (58.7%) were infected with *Plasmodium* spp., 15 (4.8%) with *Leucocytozoon* spp., and 14 (4.5%) with *Haemoproteus* spp. The most frequent lineages in *T. merula* were *P. matutinum* pLINN1 and *P. vaughani* pSYAT05, both with 97 records (31.3%) each. Other frequent lineages were *H. minutus* hTURDUS2 with 13 (4.8%), *Leucocytozoon* sp. lTUMER01 with seven (2.3%), and *P. matutinum* pAFTRU05 with five (1.6%) infected individuals. Altogether 19 lineages were detected in *T. merula*, including eight *Plasmodium*, seven *Leucocytozoon*, and four *Haemoproteus* lineages (Table 2). Of these 20 lineages, eight (TUMER12–TUMER18, TUMER20) were first found in the present study. pTUMER12 is closely associated with *P. matutinum* pLINN1, pTUMER13–pTUMER16 with *P. vaughani* pSYAT05, hTUMER17 with *H. minutus* hTURDUS2, lTUMER18 with *Leucocytozoon* sp. lTFUS14, and lTUMER20 with *Leucocytozoon* sp. lASOT06. The new lineages each differ in one bp from the already published lineages.

**Table 2:** Avian haemosporidian parasite lineages detected in the present study

	*Plasmodium*	*Leucocytozoon*	*Haemoproteus*
*Turdus merula* n = 310	*P. matutinum* pLINN1 (97) *P. vaughani* pSYAT05 (97) *P. matutinum* pAFTRU5 (5) *P.* cf. *matutinum* pTUMER12 (2) *P. elongatum* pGRW06 (1) *P.* cf. *vaughani* pTUMER13 (1), *P.* cf. *vaughani* pTUMER14 (1), *P.* cf. *vaughani* pTUMER15 (1), *P.* cf. *vaughani* pTUMER16 (1)	*L.* sp. lTUMER01 (7) *L.* sp. lASOT06 (4) *L.* sp. lNEVE01 (1) *L.* sp. lTUMER10 (1) *L.* sp. lTUMER18 (1) *L.* sp. lTUMER20 (1) *L.* sp. lTUPHI06 (1)	*H. minutus* hTURDUS2 (13) *H. brachiatus* hLK03 (1), *H. minutus* hTUCHR01 (1) *H.* cf. *minutus* hTUMER17 (1)
*Turdus philomelos* n = 36	*P. matutinum* pLINN1 (8) *P. circumflexum* pTURDUS1 (2) *P. vaughani* pSYAT05 (1) *P.* cf. *matutinum* pTUPHI08 (1) *P.* cf. *matutinum* pTUPHI09 (1)	*L.* sp. lEUSE2 (3) *L.* sp. lTUPHI10 (3) *L.* sp. lTUPHI06 (1) *L.* sp. lTUPHI11 (1) *L.* sp. lTUPHI12 (1)	*H.* sp. hTUPHI01 (4)
*Turdus pilaris* n = 2			*H. minutus* hTUCHR01 (1)

Of the 36 *T. philomelos* individuals, twelve were infected with *Plasmodium* spp. (33.3%), six with *Leucocytozoon* spp. (16.7%), and four with *Haemoproteus* spp. (11.1%). The most frequent lineages were *P. matutinum* pLINN1 with eight (22.2%), *Haemoproteus* sp. hTUPHI01 with four (11.1%), and *Leucocytozoon* sp. lTUPHI10 and *Leucocytozoon* sp. lEUSE2 with three (8.3%) positive individuals each. Altogether, eleven lineages were detected in *T. philomelos*, including each five *Plasmodium* and *Leucocytozoon* lineages, and a single *Haemoproteus* lineage (Table 2). Of these eleven lineages, five (TUPHI08–TUPHI12) were first found in the present study. pTUPHI08 and pTUPHI09 are closely associated with *P. matutinum* pLINN1, lTUPHI10 and lTUPHI11 with *Leucocytozoon* sp. lTUPHI04, and lTUPHI12 with *Leucocytozoon* sp. lEUSE2. The new lineages differ in one bp from the already published lineages, except for lTUPHI10 and lTUPHI11, which differ in three and four bp from lTUPHI04, respectively.

Of the two *T. pilaris* samples, one was infected with *H. minutus* TUCHR01.

All sequences were uploaded to NCBI GenBank under the accession numbers MT912098–MT912353, and the new lineages and data were deposited in the MalAvi database. Data on individual birds are also provided in Additional file 1.

Numbers of positive samples are indicated in brackets. The underlined lineage names indicate lineages first detected in the present study.

### *Plasmodium* lineages of Turdidae species

Based on the ML tree calculated with all *Plasmodium* sequences available in GenBank (data not shown), three *Plasmodium* clades were identified, which mainly feature lineages from Turdidae hosts. The data of all lineages clustering in these clades are shown in three separate networks, regardless if they originated from thrushes or other birds. The first network features lineages related to *P. vaughani* and *P. unalis* (pTUR1), the second lineages related to *P. matutinum* and *P. lutzi* (pTUR2), and the third lineages isolated from American Turdidae (pTUR3). The third clade is closely related to the first one. A fourth clade (pTUR4), featuring only a few lineages isolated from Turdidae birds, is shown for taxonomic reasons, which are discussed later. Detailed information on host species, countries, authors, and publications are provided in Additional file 1. The four DNA haplotype networks feature 56 *Plasmodium* lineages, which were mainly found in thrushes (Additional file 2). Another 24 lineages, 12 of which are probably specific to thrushes, did not cluster in the networks (Additional file 3). A phylogenetic tree with the lineages featured in the networks and other *Plasmodium* lineages isolated from thrushes is shown in Additional file 4.

#### *Plasmodium vaughani*/*Plasmodium unalis* clade (pTUR1)

**Fig. 1:**
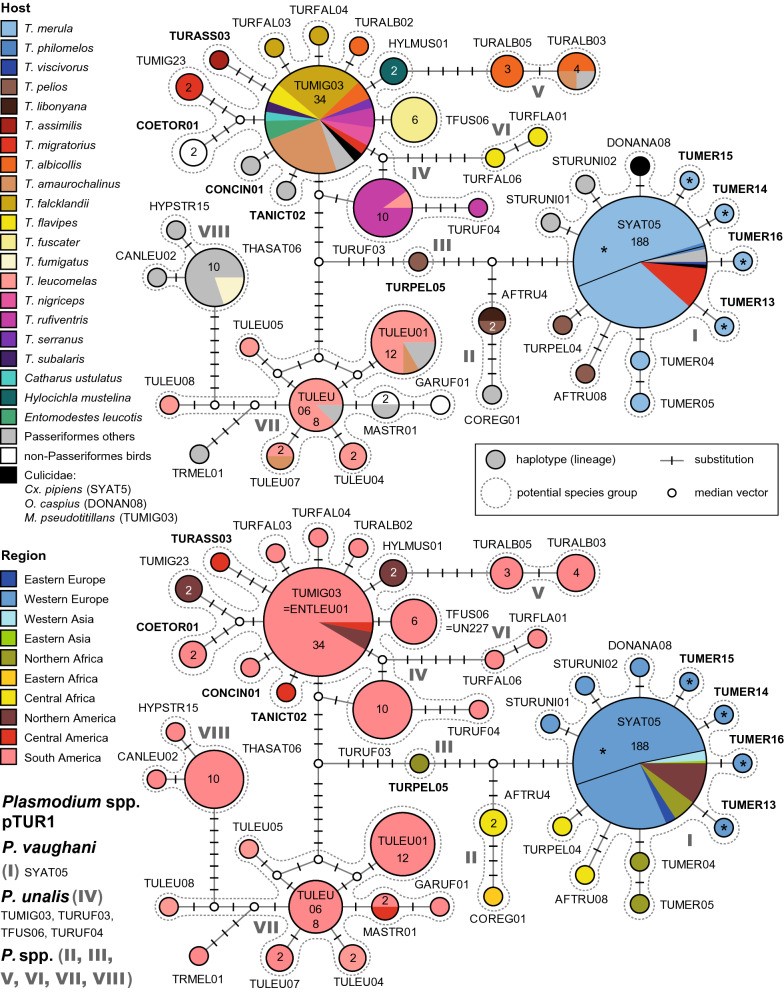
Median-Joining DNA haplotype network of partial (474 bp) *CytB* sequences of avian *Plasmodium* lineages belonging to clade pTUR1 (*P. vaughani*/*P. unalis* clade) of the subgenus *Novyella*. The upper image shows the host distribution and the lower image the geographic distribution. Asterisks mark haplotypes detected in the present study. Insert: Each circle represents a unique haplotype/lineage. The frequency of each lineage is indicated for all haplotypes with more than one record and corresponds to the size of circles. Bars on branches indicate the number of substitutions between two haplotypes. Small white circles represent median vectors, which are hypothetical (often ancestral or unsampled) sequences required to connect existing haplotypes with maximum parsimony. Groups of similar haplotypes potentially belonging to the same parasite species are framed in dotted lines and marked with Roman numbers in bold type

The network of the first major *Plasmodium* clade (Fig. 1) is reciprocally monophyletic and contains lineages attributed to *P. vaughani*, *P. unalis*, and at least six additional groups of lineages from Turdidae hosts, separated from each other by at least five bp.

The *P. vaughani* group (I) comprises the central haplotype pSYAT05 (188 records) and eleven additional, uniquely recorded lineages differing in one or two bp from the latter. pSYAT05 was almost exclusively isolated from *T. merula* in Europe (139), Morocco (10), Western Russia (5), Armenia (2), and Iran (1). It was also found in *T. philomelos* in Portugal (1) and Austria (1), *T. viscivorus* (1) in Morocco, other passeriform birds in Europe (5) and Armenia (1), as well as in *Culex pipiens* (Culicidae) in Austria (1), Turkey (1), and Japan (1). In the Americas, pSYAT05 was recorded in *T. migratorius* (19) in Michigan [24]. pSYAT05 was also recorded in *T. merula*, *T. philomelos*, and other birds in New Zealand [39, 40], but sequence data were either not present in GenBank or did not match the criteria in sequence length and quality. The new lineages pTUMER13, pTUMER14, pTUMER15, and pTUMER16 were isolated from single individuals of *T. merula* in Austria. pSTURUNI01 and pSTURUNI02 were isolated from each one specimen of *Sturnus unicolor* (Sturnidae) in Portugal, and pDONANA08 from *Ochlerotatus caspius* (Culicidae) in Spain. pTUMER04 and pTUMER05 were isolated from each one specimen of *T. merula* in Morocco, and pAFTRU08 and pTURPEL04 were isolated from each one specimen of *T. pelios* in Cameroon and Gabon, respectively.

Another group of lineages (II) includes pCOREG01 and pAFTRU4, the latter of which is connected to pSYAT05 via six substitutions. pCOREG01 was isolated from *Lamprotornis regius* (Sturnidae, 1) in Eastern Africa, and is connected to pAFTRU04 via three substitutions. pAFTRU04 was isolated from *T. pelios* (1) in Gabon and *T. libonyana* (1) in Malawi.

The haplotype pTURPEL05 (III), isolated from *T. pelios* (1) in Benin, is intermediate between the *P. vaughani* and *P. unalis* groups, separated from the first by seven and the second by eight bp.

The *P. unalis* group (IV) represents another large cluster in the network (Fig. 1), differing in at least 15 substitutions from *P. vaughani* pSYAT05. Its lineages were exclusively isolated from American birds, mostly of the family Turdidae. The central haplotype pTUMIG03 was isolated from a diverse spectrum of Turdidae hosts, from *T. falcklandii* (9) in Argentina, *T. amaurochalinus* (8), *T. albicollis* (2), *T. flavipes* (2), *T. rufiventris* (2), and *T. subalaris* (1) in Brazil, *T. nigriceps* (2), *T. serranus* (1), and *Entomodestes leucotis* (2) in Peru, and *T. migratorius* (1) and *C. ustulatus* (1) in the USA. Additional records from other passeriform birds come from *Zonotrichia capensis* (Emberizidae, 1) in Argentina, and *Tangara icterocephala* (Thraupidae, 1) in Costa Rica. The record from *Mansonia pseudotitillans* (Culicidae, 1) might indicate a competent vector [41]. The lineage pTURUF03, connected to pTUMIG03 via two substitutions (and two alternative median vectors), was isolated from *T. rufiventris* (9) and *T. leucomelas* (1) in Peru. Linked to pTURUF03 via three substitutions is pTURUF04, isolated from *T. rufiventris* (1) in Brazil. Lineage pTFUS06, differing in one bp from pTUMIG03, was isolated from *T. fuscater* (6) in Brazil. Five other lineages, differing in one bp from TUMIG03, were isolated from single birds each: pTURFAL03 and pTURFAL04 from *T. falcklandii* in Argentina, pTURALB02 from *T. albicollis* in Peru, pTANICT02 from *Tangara icterocephala* (Thraupidae) in Costa Rica, and pCONCIN01 from *Conirostrum cinereum* (Thraupidae) in Peru. pHYLMUS01, isolated from *Hylocichla mustelina* (2) in the USA, is also connected to pTUMIG03 via one substitution. The lineages pTUMIG23, isolated from *T. migratorius* (2) in the USA, and pCOETOR01 from Trochilidae (2) in Peru are connected to pTUMIG03 via three substitutions each, similar as pTURASS03 isolated from *T. assimilis* (1) in Costa Rica.

A pair of lineages comprised of pTURALB03 and pTURALB05 (V) is connected to pTUMIG03 via five substitutions. pTURALB03 was isolated from *T. albicollis* (2), *T. amaurochalinus* (1) and *Xiphocolaptes albicollis* (Dendrocolaptidae, 1) in Brazil, and pTURALB05 from *T. albicollis* (3) in Brazil as well.

Another pair of lineages, comprised of pTURFAL06 and pTURFLA01 (VI), is connected to the *P. unalis* lineages pTUMIG03 and pTURUF03 via six substitutions. Both pTURFAL06 and pTURFLA01 were isolated from single specimens of *T. flavipes* in Brazil.

Another cluster of lineages (VII), separated from the *P. unalis* group by twelve bp, was also exclusively found in the Americas. It includes pTULEU06 as central haplotype and eight additional lineages. pTULEU06 was isolated from *T. leucomelas* (7) and *Pachyramphus viridis* (Tyrannidae, 1), pTULEU01 from *T. leucomelas* (9), *T. amaurochalinus* (1), *Thamnophilus ambiguous* (Thamnophilidae, 1), and *Tyrannus melancholicus* (Tyrannidae, 1), pTULEU07 from *T. leucomelas* (1) and *T. amaurochalinus* (1), pTULEU04 from *T. leucomelas* (2), pTULEU05 from *T. leucomelas* (1), pTULEU08 from *T. leucomelas* (1), and pGARUF01 from *Galbula ruficauda* (Galbulidae, 1). All of the latter lineages were recorded exclusively in Brazil. Only pMASTR01 was isolated from *Malacoptila striata* (Bucconidae, 1) in Brazil and *Tangara icterocephala* (Thraupidae, 1) in Costa Rica.

The last group of haplotypes in the network (VIII) comprises lineages pTHASAT06, pCANLEU02, and pHYPSTR15, which were exclusively found in South America. pTHASAT06 was isolated from *T. fumigatus* (2) and birds of the Thamnophilidae (6) and Troglodytidae (1) in Brazil, and from *Pipra fasciicauda* (Pipridae, 1) in Peru. pCANLEU02 was isolated from *Cantorchilus leucotis* (Troglodytidae, 1) in Brazil, and pHYPSTR15 from *Hypocnemis striata* (Thamnophilidae, 1) in Brazil as well.

#### *Plasmodium matutinum*/*Plasmodium lutzi* clade (pTUR2)

**Fig. 2:**
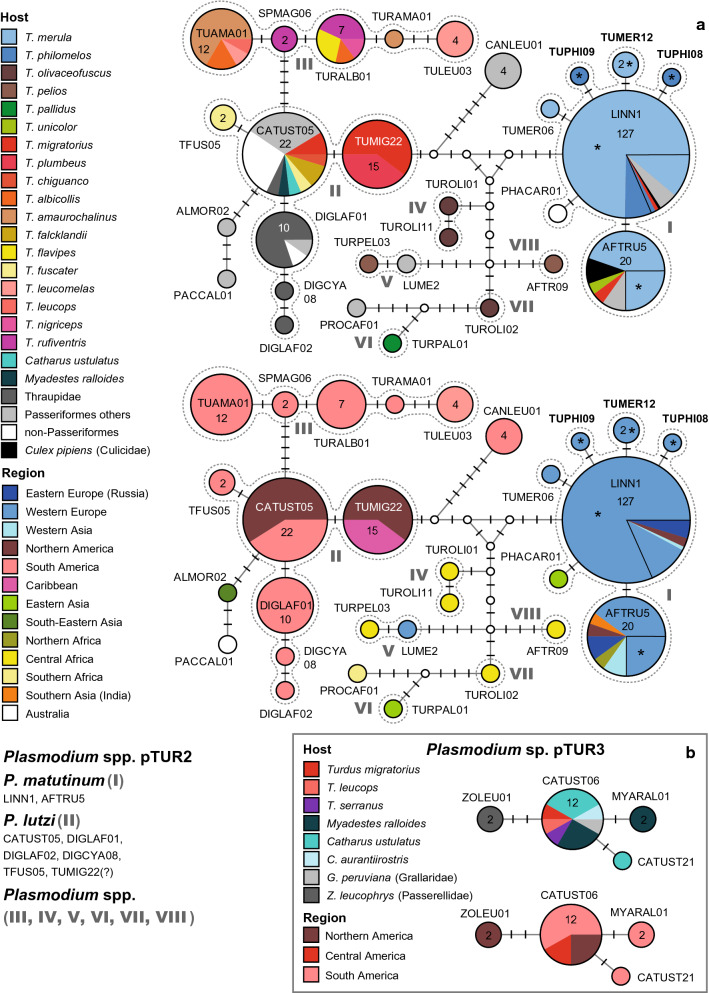
**a** *Plasmodium* lineages belonging to clade pTUR2 (*P. matutinum*/*P. lutzi* clade) of the subgenus *Haemamoeba*. **b** *Plasmodium* lineages belonging to clade pTUR3 of the subgenus *Novyella.* The upper images show the host distributions and the lower images the geographic distributions. Groups of similar haplotypes potentially belonging to the same parasite species are framed in dotted lines and marked with Roman numbers in bold type. Asterisks mark haplotypes detected in the present study

This network (Fig. 2a) includes the lineages of the second major *Plasmodium* clade with lineages linked to *P. matutinum* and *P. lutzi*. The network contains several groups of similar haplotypes/lineages showing less than three substitutions within and more than five between groups. The entire clade would feature several additional subclades with lineages of non-Turdidae birds from the Americas, which were not incorporated because the network would have become too complex.

The *P. matutinum* group (I) includes the common lineages pLINN1 (127 records) and pAFTRU5 (20) as well as five rare lineages, which have not been studied morphologically yet. pLINN1 was isolated from *T. merula* in Austria (90), Portugal (8), Western Russia (4), Hungary (4), Switzerland (3), *T. philomelos* in Austria (8), Portugal (1), and Armenia (1), *T. migratorius* (1) in the USA, other passeriform birds in Western Russia (2), Lithuania (1), Italy (1), Portugal (1), and the USA (1), and from *Cx. pipiens* (Culicidae, 1) in the USA. pAFTRU5 was isolated from *T. merula* in Austria (5), Portugal (5), Armenia (2), Russia (1), and Morocco (1), *T. migratorius* (1) in the USA, *T. unicolor* (1) in India, *Cyanistes caeruleus* (Paridae, 1) in Russia, *Corvus corone* (Corvidae, 1) in Germany, and *Cx. pipiens* (Culicidae) in Germany (1) and Austria (1). pTUMER06 was isolated from *T. merula* (1) in Portugal, and pTUMER12 from *T. merula* (2) in Austria. pTUPHI08 and pTUPHI09 were isolated from single individuals of *T. philomelos* in Austria, and PHACAR01 from *Phalacrocorax carbo* (Phalacrocoracidae, 1) in Mongolia.

The lineages of the *P. lutzi* group (II) were exclusively found in the Americas. They are separated from the *P. matutinum* group by a minimum of six substitutions. The group comprises the three common lineages pCATUST05 (22), pTUMIG22 (15), pDIGLAF01 (10), and pTFUS05 (2), and the uniquely detected lineages pDIGCYA08 and pDIGLAF02. The host composition differs strongly between lineages although they differ in one or a few bp only. pCATUST05 was found in *T. migratorius* (2) and *C. ustulatus* (1) in the USA, *T. fuscater* (1) in Colombia, *T. chiguanco* (1) and *M. ralloides* (1) in Peru, and *T. falcklandii* (2) in Argentina. Apart from Turdidae hosts, pCATUST05 was also isolated from species of the Strigidae (6), Paridae (3), and Parulidae (1) in the USA, Thraupidae (1) in Colombia, and Thamnophilidae (1) and Troglodytidae (2) in Peru. pTUMIG22 was exclusively found in *T. migratorius* (9) in the USA and *T. plumbeus* (6) in the Caribbean. pTFUS05 was isolated from *T. fuscater* (2) in Colombia. pDIGLAF01, linked to pCATUST05 via one substitution, was exclusively found in South American Thraupidae (8), Furnariidae (1), and Trochilidae (1). pDIGCYA08 and pDIGLAF02, differing from pDIGLAF01 in one and two bp, were isolated from single birds of the Thraupidae in Colombia. Two additional lineages are linked to pCATUST05 via three and five substitutions: pPACCAL01 from *Pachycephala caledonica* (Pachycephalidae, 1) in Australia, and pALMOR02 from *Alcippe morrisonia* (Pellorneidae, 1) in Myanmar.

A third group (III) comprises five similar haplotypes, which were exclusively found in South American thrushes. The lineages differ from pCATUST05 (*P. unalis*) in at least five bp. pTUAMA01 was isolated from *T. amaurochalinus* (8), *T. albicollis* (2), and *T. leucomelas* (1) in Brazil, and *T. leucops* (1) in Peru, pSPMAG06 from *T. rufiventris* (2) in Brazil, pTURALB01 from *T. rufiventris* (3), *T. flavipes* (2), *T. albicollis* (1) in Brazil, and *T. nigriceps* (1) in Peru, pTURAM01 from *T. amaurochalinus* (1) in Brazil, and pTULEU03 from *T. leucomelas* (4) in Brazil. None of the latter lineages has been linked to a morphospecies yet.

Several other lineages or pairs of lineages, none of which was linked to a morphospecies yet, were isolated from *Turdus* spp. in Africa and Eastern Asia. One of these groups (IV) comprises the lineages pTUROLI01 and pTUROLI11, which both were isolated from single specimens of *T. olivaceofuscus* in Sao Tome and Principe. pTUROLI01 and pTUROLI11 differ from each other in one bp, and at least in five bp from *P. matutinum* and *P. lutzi*. Another group (V) comprises pTURPEL03 isolated from *T. pelios* (1) in Gabon, and pLUME2 isolated from *Luscinia megarhynchos* (Muscicapidae, 1) in Sweden, differing in one bp. Other potential *Plasmodium* species groups comprise single lineages isolated from Eastern Asian and African *Turdus* species: pTURPAL01 (VI) was isolated from *T. pallidus* (1) in Japan, pTUROLI02 (VII) from *T. olivaceofuscus* (1) in Sao Tome and Principe, and pAFTR09 (VIII) from *T. pelios* (1) in Cameroon. The latter lineages are similar but differ in at least five bp from each other. The network includes four other lineages, which were found in non-Turdidiae hosts, pCANLEU01 from Troglodytidae (3) and Tyrannidae (1) in Brazil, and pPROCAF01 from *Promerops cafer* (Promeropidae, 1) in South Africa.

#### *Plasmodium* sp. (pTUR3)

The third network (Fig. 2b) contains lineages of a clade, which is closely related to the *P. vaughani*/*P. unalis* clade. The clade pTUR3 comprises four haplotypes differing in one to three bp, which have been found exclusively in the Americas. pCATUST06 was isolated from *C. ustulatus* in the USA (2), Colombia (1) and Costa Rica (1), *M. ralloides* (3) in Peru, *C. aurantiirostris* (1) in Costa Rica, *T. migratorius* (1) in the USA, *T. serranus* (1) and *T. leucops* (1) in Peru, and *Grallaricula peruviana* (Grallariidae, 1) in Peru. pCATUST21 was isolated from *C. ustulatus* (1) in Colombia and pMYARAL01 from *M. ralloides* (2) in Peru. The fourth lineage pZOLEU01 was isolated from *Zonotrichia leucophrys* (Passerellidae, 2) in the USA. None of these lineages have been linked to a morphospecies yet.

#### *Plasmodium homopolare* clade (pTUR4)

**Fig. 3:**
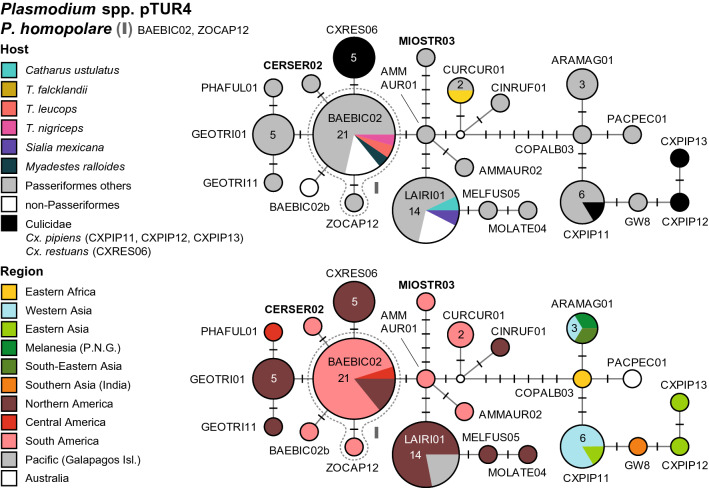
Median-Joining DNA haplotype network of partial (474 bp) *CytB* sequences of avian *Plasmodium* lineages belonging to clade pTUR4 (*P. homopolare* clade) of the subgenus *Novyella*. The upper image shows the host distribution and the lower image the geographic distribution. Groups of similar haplotypes potentially belonging to the same parasite species are framed in dotted lines and marked with Roman numbers in bold type

Another *Plasmodium* clade (Fig. 3) comprises lineages attributed to *P. homopolare* (pBEABIC02, pZOCAP02) and several additional ones. The network includes two sequence clusters separated by seven or more bp, the first with 16 lineages isolated from birds in the Americas and the second with seven lineages isolated from birds in Asia. The network would contain several additional lineages (pDENPET02, pELALB02, pGEOTRI03, pGEOTRI05, pGW4, pGW6, pSEIAUR02, pSERUT09, pTABI07, pTROAED21, pTUMIG02, and pZOCAP11), for which only shorter *CytB* fragments were available. The American lineages were isolated from an extremely wide host range including passeriform (Emberizidae, Icteridae, Mimidae, Paridae, Parulidae, Pipridae, Sturnidae, Thraupidae, Thamnophilidae, Troglodytidae, and Turdidae), apodiform, galliform, and strigiform birds. Three of the lineages were isolated also from Turdidae species: pBAEBIC02 was isolated from each one individual of *M. ralloides*, *T. leucops*, and *T. nigriceps* in Peru, pLAIRI01 from *C. ustulatus* (1) and *S. mexicana* (1) in the USA, and pCURCUR01 from *T. falcklandii* (1) in Argentina. The group of Asian lineages is separated from the group of American lineages by at least six bp. The Asian lineages were isolated from birds of the families Fringillidae, Muscicapidae, Nectariniidae, Phylloscopidae, Pachycephalidae, Parulidae, and Thraupidae. Detailed information on host species, localities, and lineages is provided in Additional file 1.

#### Rare *Plasmodium* lineages from Turdidae birds

Several other *Plasmodium* lineages were also recorded from birds of the Turdidae but did not cluster within the four clades shown as DNA haplotype networks. Some of these lineages were isolated from single individuals of thrushes, whereas others were predominantly found in other passeriform and non-passeriform birds. pALEDIA01 was isolated from *C. occidentalis* and *T. assimilis* in Mexico, pBT7 (*P.* cf. *circumflexum*) from *C. ustulatus* and *T. migratorius* in Alaska and *C. ustulatus* in Costa Rica, pCORPIL01 from *T. rufiventris* in Brazil, pDENPET03 (*P. nucleophilum*) from *T. migratorius* in the USA, *T. hauxwelli* in Peru, and *T. rufiventris* in Brazil, TSUB01 (*Plasmodium* cf. *juxtanucleare*) from *T. subularis* in Brazil, pGLYSPI06 and pTURAMA03 from *T. amaurochalinus* in Brazil, pGRW06 (*P. elongatum*) from *T. albicollis* and *T. leucomelas* in Brazil, *T. merula* in Austria, and *T. merula* and *T. philomelos* in New Zealand [40, 42], pLEPCOR04 from *T. hauxwelli* in Peru, pMYRHEM02 from *T. albicollis* in Brazil, pMYRLEU01 from *T. hauxwelli* in Peru, pPADOM09 from *C. aurantiirostris* in Costa Rica, pPADOM11 (*P.* cf. *elongatum*) from *T. fumigatus* and *T. migratorius* in the USA, pSEIAUR01 (*P. cathemerium*) from *C. ustulatus* and *T. migratorius* in the USA, pSGS1 (*Plasmodium relictum*) from *T. viscivorus* in Morocco, pTRMUS02 (*P.* cf. *elongatum*), TULEU02 and VOLJAC02 from *T. leucomelas* in Brazil, pTUMIG1 from *T. migratorius* in the USA, TUROLI03 (*P.* cf. *elongatum*), pTUROLI04 and pTUROLI12 from *T. olivaceofuscus* in Sao Tome and Principe, and TURDUS1 (*P. circumflexum*) and pBAFLA04 from *T. philomelos* in Austria and Sweden, respectively (see for details in Additional file 1). A phylogenetic tree with all *Plasmodium* lineages isolated from Turdidae hosts (and related lineages included in the networks) is provided in Additional file 4. A summary of these *Plasmodium* lineages is provided in Additional file 3, which also includes information on the main host groups (bird families).

### *Haemoproteus* lineages of Turdidae birds

**Fig. 4:**
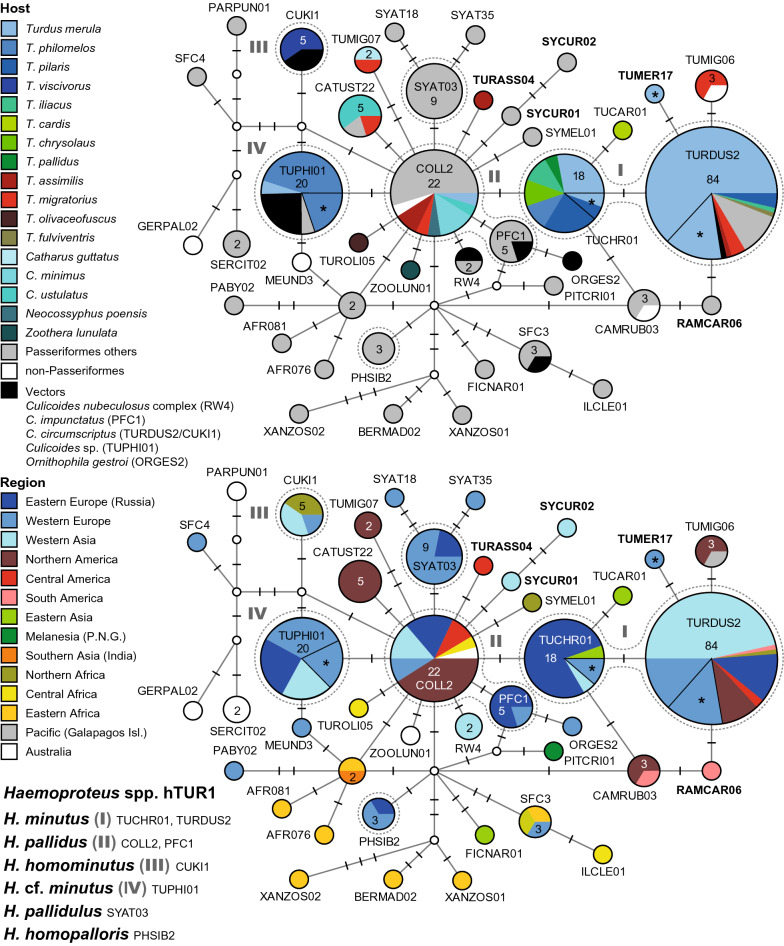
Median-Joining DNA haplotype network of partial (474 bp) *CytB* sequences of avian *Haemoproteus* lineages belonging to clade hTUR1 (*H. minutus* group). The upper image shows the host distribution and the lower image the geographic distribution. Groups of similar haplotypes potentially belonging to the same parasite species are framed in dotted lines and marked with Roman numbers in bold type. Asterisks mark haplotypes detected in the present study

**Fig. 5:**
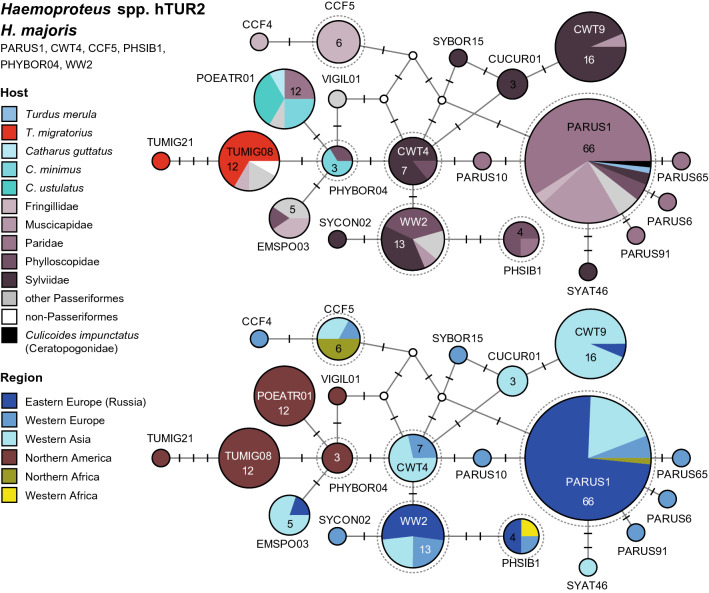
Median-Joining DNA haplotype network of partial (474 bp) *CytB* sequences of avian *Haemoproteus* lineages belonging to clade hTUR2 (*H. majoris* group). The upper image shows the host distribution and the lower image the geographic distribution. Groups of similar haplotypes potentially belonging to the same parasite species are framed in dotted lines and marked with Roman numbers in bold type

There is only a single clade (hTUR1, Fig. 4) featuring multiple *Haemoproteus* lineages, which are frequently found in birds of the Turdidae. Besides, also the *Haemoproteus majoris* clade (hTUR2, Fig. 5) comprises four lineages from North American thrushes, which are not linked to morphospecies yet. Several further lineages attributed or similar to other *Haemoproteus* species, were found in single specimens of the Turdidae, but are common in other passeriform birds. Detailed information on host species, countries, authors, and publications is provided as Additional file 1. The two DNA haplotype networks feature 17 *Haemoproteus* lineages, which were mainly found in thrushes (Additional file 5). Another 20 lineages, 11 of which are probably specific to thrushes, did not cluster in the networks (Additional file 6). A phylogenetic tree with the lineages featured in the networks and other *Haemoproteus* lineages isolated from thrushes is shown in Additional file 7.

#### *Haemoproteus minutus* clade (hTUR1)

This network features several *Haemoproteus* lineages, which are common in birds of the Turdidae (Fig. 4). It also contains more than 20 further lineages, which were mostly isolated from other passeriform birds and not discussed here. Differently than in the *Plasmodium* networks, lineages linked to different *Haemoproteus* species differ only in one to six bp from each other in this network.

The *H. minutus* group (I) currently includes the lineages hTURDUS2 (84 records) and hTUCHR01 (18). Three other lineages from *Turdus* spp. differ by one bp from the latter two lineages, but have not been confirmed morphologically as *H. minutus* yet: hTUMIG06, hTUCAR01, and hTUMER17. hTURDUS2 was isolated from *T. merula* in Armenia (34), Austria (13), Lithuania (5), Portugal (5), Western Russia (5), Iran (2), and Morocco (1), and from *T. pilaris* (3) and *T. iliacus* (1) in Russia. Other records come from *Erithacus rubecula* (Muscicapidae, 1) and *Garrulus glandarius* (Corvidae, 1) in Armenia, *Muscicapa striata* (Muscicapidae, 1) in Western Russia, and *Culicoides circumscriptus* (Ceratopogonidae, 1) in Turkey. In the Americas, hTURDUS2 was isolated from *T. migratorius* (3) in the USA, *T. fulviventris* (1) in Peru, and *T. assimilis* (1) in Costa Rica. Moreover, hTURDUS2 was isolated from *Loxia leucoptera* (Fringillidae, 3), *Cardellina pusilla* (Parulidae, 1) and *Setophaga striata* (Parulidae, 1) in the USA, and *Tangara icterocephala* (Thraupidae, 1) in Costa Rica. The lineage hTUCHR01 was isolated from *T. merula* in Western Russia (4), Austria (1), and Armenia (1), *T. chrysolaus* (2) in Eastern Russia, *T. pilaris* in Russia (4) and Austria (1), *T. philomelos* (2) in Russia, *T. iliacus* (2) in Russia, and *T. pallidus* (1) in Japan. The yet unlinked lineage hTUMIG06 was isolated from *T. migratorius* (2) in the USA and *Spheniscus mendiculus* (Spheniscidae, 1) from the Galapagos Islands, hTUMER17 from *T. merula* (1) in Austria, and hTUCAR01 from *T. cardis* (1) in South Korea.

The *Haemoproteus pallidus* group (II) is connected to hTUCHR01 (*H. minutus*) via one substitution and includes the lineages hCOLL2 (22) and hPFC1 (5). hCOLL2 was isolated from *T. assimilis* (2) in Costa Rica, *C. minimus* (3), *C. ustulatus* (1), and *T. migratorius* (1) in the USA, *T. merula* (1) in Western Russia, and *Neocossyphus poensis* (1) in Cameroon. Other records come from Muscicapidae in Hungary (1), Sweden (1), and Western Russia (1), Sylviidae in Armenia (2) and Western Russia (1), *Cyanistes caeruleus* (Paridae, 1) in Western Russia, *Garrulus glandarius* (Corvidae, 1) in Armenia, *Empidonax alnorum* (Tyrannidae, 1), *Setophaga coronata* (Parulidae, 1), *Phylloscopus borealis* (Phylloscopidae, 1), and *Calidris minutilla* (Scolopacidae, 1) in Alaska (USA), and *Scenopoeetes dentirostris* (Ptilonorhynchidae, 1) in Australia. Most records of hCOLL2 were isolated from the two Muscicapidae species *Ficedula albicollis* and *F. hypoleuca*, but these data are only available in the MalAvi database and were not included. The second lineage hPFC1 was isolated from *F. hypoleuca* in Western Russia (3) and Sweden (1), and *Culicoides impunctatus* (Ceratopogonidae, 1) in Western Russia. The latter was shown to be a competent vector for lineage hPFC1 [43]. The network contains several additional haplotypes connected to hCOLL2 via one or two substitutions, which were isolated from birds of the Turdidae, but have not been linked to morphospecies yet: hTURASS04 from *T. assimilis* (1) in Costa Rica, hTUROLI05 from *T. olivaceofuscus* (1) in Sao Tome and Principe, hZOOLUN01 from *Zoothera lunulata* (1) in Australia, hTUMIG07 from *T. migratorius* (1) and *C. guttatus* (1) in the USA, and hCATUST22 from *C. ustulatus* (3), *T. migratorius* (1), and *Melospiza lincolnii* (Passerellidae, 1) in the USA.

*Haemoproteus homominutus* is represented by the lineage hCUKI1 (III), which is separated from both hTUPHI01 and hCOLL2 by two bp. hCUKI1 was isolated from *T. viscivorus* in Lithuania (1) and Morocco (2), and *Culicoides circumscriptus* (Ceratopogonidae, 2) in Turkey.

Another frequent haplotype, hTUPHI01 (IV), was linked to *H. minutus* by [44]. It differs from hCOLL2 (*H. pallidus*) in one bp, and from hTUCHR01 and hTURDUS2 (*H. minutus*) in two and three bp, respectively. hTUPHI01 was isolated from *T. philomelos* in Austria (4), Russia (4), Armenia (3), Sweden (1), and Bulgaria (1), *T. merula* (1) in Western Russia, and *Garrulus glandarius* (1) in Armenia. In Germany, hTUPHI01 was isolated from *Culicoides* sp. (5).

*Haemoproteus pallidulus* was not found in Turdidae birds but is mentioned here because it is part of network hTUR1. It includes only the lineage hSYAT03, which differs from hCOLL2 (*H. pallidus*) by one bp. hSYAT03 was exclusively isolated from *Sylvia atricapilla* (Sylviidae) in Portugal (5), Spain (1), Western Russia (2), and Sweden (1). *Haemoproteus homopalloris* was also not found in Turdidae. This species is represented by the lineage hPHSIB2, which differs from hCOLL2 and hTUPHI01 in two bp. hPHSIB2 was isolated from *Phylloscopus sibilatrix* (Phylloscopidae) in Western Russia (1) and Lithuania (1), and *Hippolais polyglotta* (Acrocephalidae) in Spain (1).

#### *Haemoproteus majoris* clade (hTUR2)

Another *Haemoproteus* clade (Fig. 5) comprises several lineages attributed to *Haemoproteus majoris*, which were mainly isolated from non-Turdidae Passeriformes in Europe, Western Asia, and Africa. However, five similar lineages were isolated from birds in North America, mostly from Turdidae. These North American lineages differ in one or a few bp from lineages attributed to *H. majoris*. According to [45], *H. majoris* comprises the lineages hPARUS1 (66), hWW2 (13), hCWT4 (7), hCCF5 (6), and hPHISIB1 (4). However, several similar lineages in this clade are not linked to morphospecies yet. The group of lineages isolated from North American birds includes hTUMIG08 (12), hPOEATR01 (12), hPHYBOR04 (3), and hVIGIL01 (1). hTUMIG08 was isolated from *T. migratorius* (8), *Regulus calendula* (Regulidae, 1), *Empidonax hammondii* (Tyrannidae, 1), *Acanthis flammea* (Fringillidae, 1), and *Picoides dorsalis* (Picidae, 1). hPOEATR01 was isolated from *C. ustulatus* (4), *C. minimus* (3), *C. guttatus* (1), *Poecile hudsonicus* (Paridae, 2), *Poecile atricapillus* (Paridae, 1), and *Pica hudsonica* (Picidae, 1), hPHYBOR04 from *C. minimus* (2) and *Phylloscopus borealis* (Phylloscopidae, 1), hTUMIG21 from *T. migratorius* (1), and hVIGIL01 from *Vireo gilvus* (Vireonidae, 1) in the USA. hEMSPO03 is linked to hPHYBOR04 by one substitution and was found in passeriform birds in Armenia (4) and Russia (1).

#### Additional *Haemoproteus* lineages from Turdidae birds

Additional *Haemoproteus* lineages not represented in the networks were isolated from birds of the Turdidae, mainly in the Americas. hAFR130 was isolated from *Geokichla gurneyi* in Malawi. hCATGUT01, isolated from *C. guttatus* in the USA, is similar to lineages of *Haemoproteus platalae* from North American anseriform and galliform birds and to *Haemoproteus enucleator* from Central African coraciiform birds. hCATUST07, hCATUST10, hCATUST15, hCATUST16, hCATUST17, hCATUST18, and hCATUST19 were isolated from *C. ustulatus* in the USA. hCATUST15 and hCATUST17 are similar to hZOSXAN03 and hZOSLAT10 (*Haemoproteus killangoi*), hCATUST16, hCATUST18, and hVIGIL09 are similar to hVIOLI06 (*Haemoproteus vireonis*) from South American Vireonidae, and hCATUST19 differs in one bp from hSISKIN01 (*Haemoproteus tartakovskyi*). hCHRKLA01, isolated from *T. libonyana* in South Africa, is similar to hPLOMEL01 and hPLOMEL02 (*H. homobelopolskyi*). hCOLPAS04 and hCYCYAN01 were isolated from *T. assimilis* in Costa Rica, whereby hCOLPAS04 was found primarily in Columbidae and Tyrannidae in South America, and hCYCYAN01 in Thraupidae in the Americas. The lineage hDUNNO01 was isolated from *Ixoreus naevius* in the USA, hLK03 (*Haemoproteus brachiatus*) from *T. merula* in Austria, hSIAMEX01 from *Sialia mexicana* in the USA, hSPIARB01 from *C. ustulatus* in the USA, hTROAED20 (*Haemoproteus witti*) from *M. ralloides*, *T. serranus* and *T. nigriceps* in Ecuador, hTURUF02 from *T. rufiventris* in Brazil, hVIGIL09 from *T. nigriceps* in Peru, and hZOCAP14 (*Haemoproteus* cf. *erythrogravidus*) from *C. fuscater* in Peru. A phylogenetic tree with all *Haemoproteus* lineages isolated from Turdidae hosts (and related lineages included in the network) is provided in Additional file 7. A summary of these *Haemoproteus* lineages is provided in Additional file 6, which also includes information on the main host groups (bird families).

### *Leucocytozoon* lineages of Turdidae birds

Altogether, eight *Leucocytozoon* clades were identified, which almost exclusively comprise lineages isolated from birds of the Turdidae. The lineages of some clades (lTUR2, lTUR3, lTUR4, lTUR5) were almost exclusively isolated from *Turdus* spp., whereas those of others (lTUR6, lTUR7, lTUR8) were predominantly isolated from *Catharus* spp. The network of clade lTUR1 contains lineages isolated from both *Turdus* spp. and *Catharus* spp., however, these differ in several bp from each other. Detailed information on host species, countries, authors, and publications are provided in the Additional file 1, and a phylogenetic tree is shown in Additional file 8. The eight DNA haplotype networks feature 94 *Leucocytozoon* lineages, which were mainly found in thrushes (Additional file 9). Another 25 lineages, 18 of which are probably specific to thrushes, did not cluster in the networks (Additional file 10). A phylogenetic tree with the lineages featured in the networks and other *Haemoproteus* lineages isolated from thrushes is shown in Additional file 8. The lineages included in the networks have not been linked to a morphospecies yet, except for four parasite lineages studied by [16] in North American birds from Alaska. They identified four parasite lineages in thrushes (lCATGUT02, lTUMIG15, and lTUMIG11), which matched *L. majoris* morphotypes, and one lineage (lCATMNI01), which matched *L. dubreuili* morphotypes (Fig. 1 and Additional file 2 in [16]). The three *L. majoris*-like lineages differ by 4.6 to 10.3% (*p*-distance) and cluster in three different clades/networks in the present study (lTUR1, lTUR2, and lTUR7). They were also classified as separate species in the multi-gene species delimitation analysis of [16].

#### *Leucocytozoon* spp. lTUR1

**Fig. 6:**
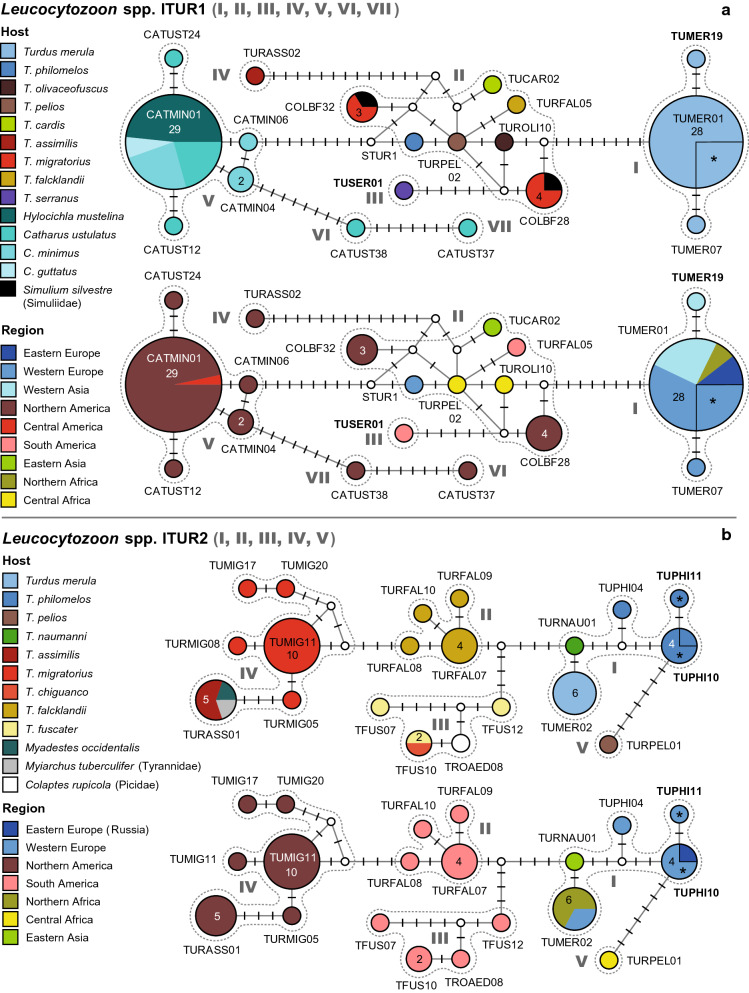
Median-Joining DNA haplotype network of partial (474 bp) *CytB* sequences of avian *Leucocytozoon* lineages belonging to clades lTUR1 (**a**) and lTUR2 (**b**). The upper images show the host distributions and the lower image the geographic distributions. Groups of similar haplotypes potentially belonging to the same parasite species are framed in dotted lines and marked with Roman numbers in bold type. Asterisks mark haplotypes detected in the present study

This network (Fig. 6a) features three groups of lineages separated by eight or more substitutions from each other, and a unique haplotype separated from the central group by five bp.

The first group (I) includes lTUMER01 (28) as a central haplotype, to which lTUMER07 (1) and lTUMER019 (1) are linked via two substitutions each. lTUMER01 was exclusively isolated from *T. merula* in Portugal (9), Austria (7), Armenia (7), Western Russia (3), and Morocco (2). The lineages lTUMER07 and lTUMER019 were isolated from each one individual of *T. merula* in Portugal and Armenia, respectively.

The second group (II) includes the lineages lCOLBF28 (4), lCOLBF32 (3), lSTUR1 (1), lTUCAR2 (1), lTURFAL05 (1), lTURPEL02 (1), and lTUROLI10 (1), separated by one to four bp from each other. lCOLBF28 was isolated from *T. migratorius* (3) and *Simulium silvestre* (Simuliidae, 1) in the USA, and lCOLBF32 from *T. migratorius* (2) and *Simulium silvestre* (1) in the USA as well. lSTUR1 was isolated from *T. philomelos* (1) in Portugal, lTUCAR02 from *T. cardis* (1) in Japan, lTURPEL02 from *T. pelios* (1) in Gabon, and lTUROLI10 from *T. olivaceofuscus* (1) in Sao Tome and Principe.

Two lineages are attached to group II: lTUSER01 (group III), isolated from *T. serranus* (1) in Peru and separated via five substitutions, and lTURASS02 (group IV), isolated from *T. assimilis* (1) in Mexico, and separated via 15 substitutions.

The fifth group (V) includes the lineages lCATMIN01 (29), lCATMIN04 (2), lCATUST12 (1), lCATUST24 (1), and lCATMIN06 (1), which were isolated from *Catharus* spp., almost exclusively in North America. The central haplotype lCATMIN01 was isolated from *Hylocichla mustelina* (13), *C. minimus* (7), *C. ustulatus* (6), and *C. guttatus* (2) in the USA, and *H. mustelina* (1) in Honduras. lCATUST12 and lCATUST24 were isolated from each one specimen of *C. ustulatus*, lCATMIN06 from *C. minimus* (1), and lCATMIN04 from *C. minimus* (2).

Two haplotypes, lCATUST38 (group VI) and lCATUST37 (group VII), are connected to group V via eight and 15 bp, respectively. Both lineages were isolated from single specimens of *C. ustulatus* in the USA.

#### *Leucocytozoon* spp. lTUR2

The network of this *Leucocytozoon* clade (Fig. 6b) includes five groups of haplotypes, which differ by a minimum of four substitutions from each other and show unique geographic distributions. The first group (I) includes the lineages lTUPHI10 (4), lTUPHI11 (1), lTUPHI04 (1), lTURNAU01 (1), and lTUMER02 (6). lTUPHI10 was isolated from *T. philomelos* in Austria (3) and Western Russia (1), lTUPHI04 from *T. philomelos* (1) in Portugal, and lTUPHI11 from *T. philomelos* (1) in Austria. lTUMER02 was isolated from *T. merula* in Morocco (4), Portugal (1), and the Azores (1), and lTURNAU01 from *T. naumanni* (1) in Japan.

The second group (II) is separated from groups I and III by four substitutions and comprises four similar haplotypes, which were exclusively isolated from *T. falcklandii* in Argentina: lTURFAL07 (4), lTURFAL08 (1), lTURFAL09 (1), and lTURFAL10 (1).

The third group (III) comprises four haplotypes, which were all found in South American birds: lTFUS10 (2), lTFUS07 (1), lTFUS12 (1), and lTROAED02 (2). lTFUS10 was isolated from *T. chiguanco* (1) in Peru and *T. fuscater* (1) in Colombia. lTFUS07 and lTFUS12 were isolated from single specimens of *T. fuscater* in Colombia. lTROAED08 was isolated from a specimen of *Colaptes rupicola* (Picidae) in Peru.

The fourth group (IV) includes five lineages isolated from North American birds: lTUMIG11 (10), lTURASS01 (5), lTUMIG20 (1), lTURMIG05 (1), and lTURMIG08 (1). lTUMIG11 was isolated exclusively from *T. migratorius* (10) in the USA. lTURASS01 was isolated from *T. assimilis* (3) and *M. occidentalis* (1) in Mexico, and *Myiarchus tuberculifer* (Tyrannidae, 1) in the USA. lTURMIG05, lTURMIG08, lTUMIG17, and lTUMIG20 were all isolated from single specimens of *T. migratorius* in the USA.

The lineage lTURPEL01 (group V) is connected to lTUPHI10 (group I) via six substitutions. It was isolated from a specimen of *T. pelios* in Equatorial Guinea.

#### *Leucocytozoon* spp. LTUR3

**Fig. 7:**
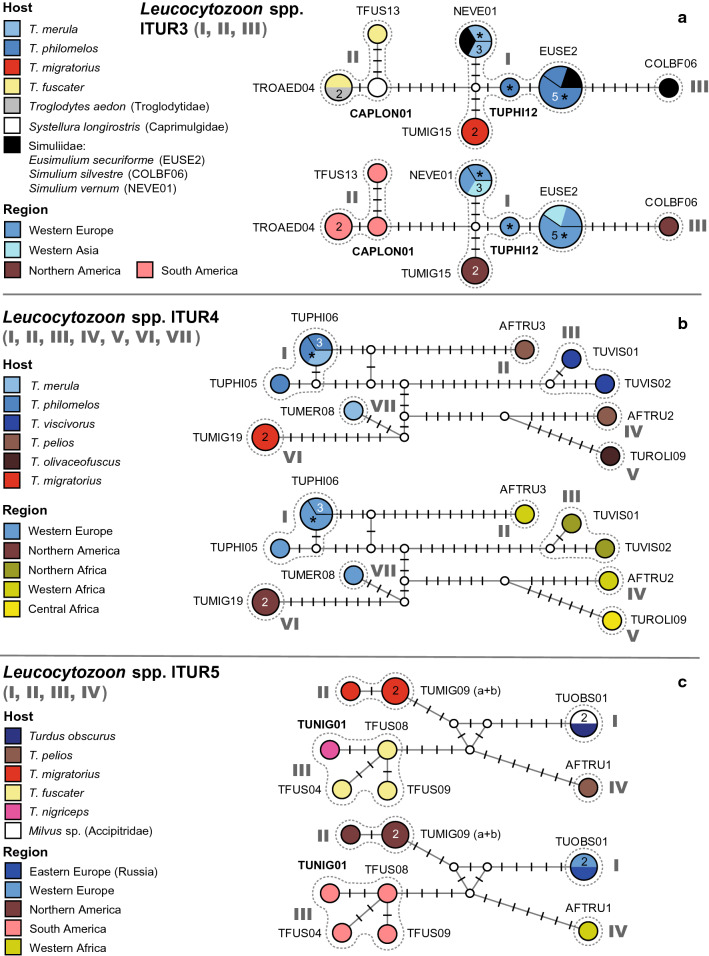
Median-Joining DNA haplotype network of partial (474 bp) *CytB* sequences of avian *Leucocytozoon* lineages belonging to clades lTUR3 (**a**), lTUR4 (**b**), and lTUR5 (**c**). The upper images show the host distributions and the lower image the geographic distributions. Groups of similar haplotypes potentially belonging to the same parasite species are framed in dotted lines and marked with Roman numbers in bold type. Asterisks mark haplotypes detected in the present study

This network (Fig. 7a) comprises a central group of lineages and two further groups, separated by at least six substitutions. The first group (I) includes four lineages connected via one to four substitutions: lEUSE02 (5), lNEVE01 (3), lTUPHI12 (1), and lTUMIG15 (2). lEUSE2 was isolated from *T. philomelos* in Austria (3) and Turkey (1), and *Eusimulium securiforme* (Simuliidae, 1) in Czechia. lTUPHI12 was isolated from *T. philomelos* (1) in Austria, and lTUMIG15 from *T. migratorius* (2) in Alaska. lNEVE01 was isolated from *T. merula* in Austria (1) and Czechia (1), and *Aegolius funereus* (1) in Czechia. The second group (II) includes three lineages obtained from South American birds: lTROAED04 (2), lTFUS13 (1), and lCAPLON01 (1). lTROAED04 was isolated from *T. fuscater* (1) and *Troglodytes aedon* (1) in Peru, lTFUS13 from *T. fuscater* (1) in Colombia, and lCAPLON01 from *Caprimulgus longirostris* (1) in Peru. The third group (III) is separated from group I by six substitutions and contains only the lineage lCOLBF06, isolated from one specimen of *Simulium silvestre* (Simuliidae) in the USA (1). Potential bird hosts of lineage lCOLBF06 have not been identified yet.

#### *Leucocytozoon* spp. lTUR4

This network (Fig. 7b) features seven groups of distantly related, rarely recorded lineages, which differ from each other by twelve or more bp. The first group (I) includes two haplotypes connected via two substitutions: lTUPHI06 (3) and lTUPHI05 (1). lTUPHI06 was isolated from *T. philomelos* in Austria (1) and Portugal (1), and *T. merula* (1) in Austria. The second group (II) includes only lineage lAFTRU3, isolated from *T. pelios* (1) in Nigeria. The third group (III) includes two lineages connected via four substitutions: lTUVIS01 and lTUVIS02, which both were isolated from single specimens of *T. viscivorus* in Morocco. The fourth group (IV) includes lAFTRU2 isolated from *T. pelios* (1) in Nigeria. The fifth group (V) includes lTUROLI01 isolated from *T. oliaceofuscus* (1) in Sao Tome and Principe. Group six (VI) includes lTUMIG19 isolated from *T. migratorius* (2) in the USA, and group seven (VII) includes lTUMER08 isolated from *T. merula* (1) in Portugal.

#### *Leucocytozoon* spp. lTUR5

This network (Fig. 7c) features four groups of lineages separated by a minimum of eight substitutions from each other. The first group (I) includes lTUOBS01 isolated from *Turdus obscurus* (1) in Western Russia and *Milvus* sp. (1) in Spain. The second (II) includes two lineages, lTUMIG09a and lTUMIG09b [differing in the last bp of the alignment], isolated from *T. migratorius* (3) in Alaska. The third group (III) includes three lineages (lTFUS04, lTFUS08, and lTFUS09) isolated from single individuals of *T. fuscater* in Colombia, whereas lTUNIG01 was isolated from *T. nigriceps* (1) in Peru. The fourth group (IV) is represented by a single lineage, lAFTRU01, recorded from *T. pelios* (1) in Nigeria.

#### *Leucocytozoon* spp. lTUR6

**Fig. 8:**
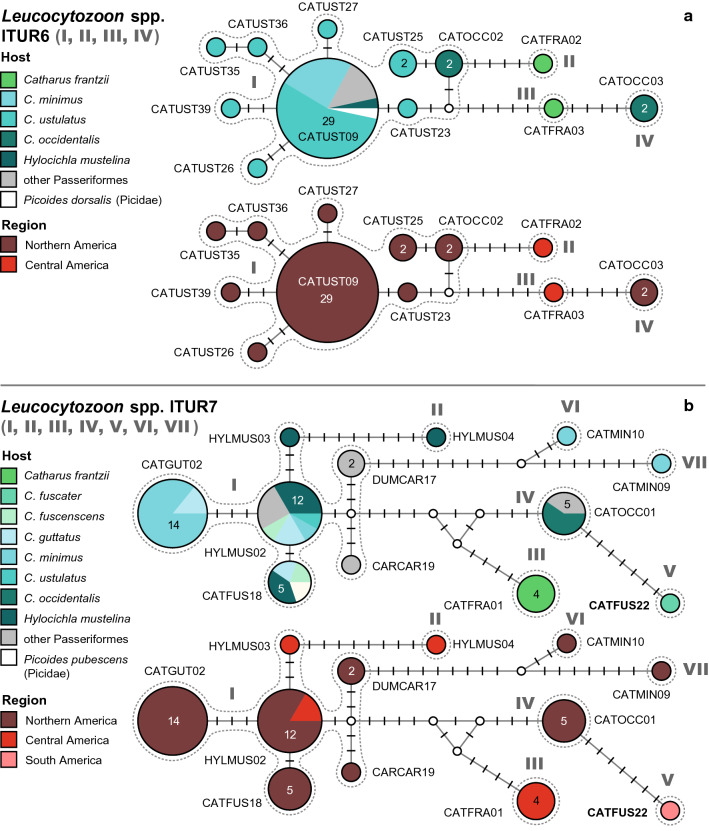
Median-Joining DNA haplotype network of partial (474 bp) *CytB* sequences of avian *Leucocytozoon* lineages belonging to clades lTUR6 (**a**) and lTUR7 (**b**). The upper images show the host distributions and the lower image the geographic distributions. Groups of similar haplotypes potentially belonging to the same parasite species are framed in dotted lines and marked with Roman numbers in bold type

This network (Fig. 8a) features four groups of lineages separated by a minimum of four bp. The lineages were almost exclusively isolated from *Catharus* spp. in Northern and Central America. The first group (I) comprises nine lineages: lCATUST09 (29), lCATOCC02 (2), lCATUST25 (2), lCATUST23 (1), lCATUST26 (1), lCATUST27 (1), lCATUST35 (1), lCATUST36 (1), and lCATUST39 (1). lCATUST09 was isolated from *C. ustulatus* (16), *C. minimus* (7), *H. mustelina* (1), *Melospiza lincolnii* (Fringillidae, 1), *Poecile hudsonica* (Paridae, 1), *Phylloscopus borealis* (Sylviidae, 1), *Empidonax alnorum* (Tyrannidae, 1), and *Picoides dorsalis* (Picidae, 1) in the USA. lCATOCC02 was isolated from *C. occidentalis* (2) in Mexico, and lCATUST25 from *C. ustulatus* (2) in the USA. The other six lineages, lCATUST23, lCATUST25, lCATUST26, lCATUST27, lCATUST35, lCATUST36, and lCATUST39 were all isolated from single specimens of *C. ustulatus* in the USA. The second group (II), attached to group I via four substitutions, includes only lCATFRA02 isolated from *Catharus franzii* (1) in Nicaragua. The third group (III), attached to group I and IV by six and four bp, respectively, includes lCATFRA03, isolated from *Catharus franzii* (1) in Nicaragua as well. The fourth group (IV) features the lineage lCATOCC03 isolated from *C. occidentalis* (2) in Mexico.

#### *Leucocytozoon* spp. lTUR7

This network (Fig. 8b) features seven groups of haplotypes separated by seven of more substitutions from each other. Apart from the first group, all others comprise single lineages. The first group (I) comprises the lineages lCATGUT02 (14), lHYLMUS02 (12), lCATFUS18 (5), lHYLMUS03 (1), lDUMCAR17 (2), and lCARCAR19 (1). lCATGUT02 was isolated from *C. minimus* (12) and *C. guttatus* (2) in the USA. lHYLMUS02 was isolated from *H. mustelina* in the USA (2), Nicaragua (1), and Honduras (1), and from *C. guttatus* (2), *C. minimus* (1)*, C. ustulatus* (1)*, C. fuscescens* (1), *Dumetella carolinensis* (Mimidae, 1), *Poecile carolinensis* (Paridae, 1), and *Troglodytes aedon* (Troglodytidae, 1) in the USA. lCATFUS18 was isolated from *H. mustelina* (2), *C. guttatus* (1), *C. fuscescens* (1), and *Picoides pubescens* (Picidae, 1) in the USA. lHYLMUS03 was isolated from *H. mustelina* (1) in the Nicaragua, lDUMCAR17 from *Cardinalis cardinalis* (Fringillidae, 1) and *D. carolinensis* (1) in the USA, and lCARCAR19 from *C. cardinalis* (1) in the USA. The lineage lHYLMUS04 (group II), separated from group I by eight bp, was isolated only from *H. mustelina* (1) in Nicaragua. lCATFRA01 (group III), separated from groups I and IV by ten and eight bp, respectively, was solely isolated from *Catharus frantzii* (4) in Nicaragua. lCATOCC01 (group IV) is separated from groups I, III and V by at least seven bp. It was isolated from *C. occidentalis* (3) and *Atlapetes pileatus* (Emberizidae, 2) in Mexico. lCATFUS22 (group V), linked to lCATOCC01 (group IV) via seven substitutions, was isolated from *C. fuscater* (1) in Peru. lCATMIN09 (group VI) and lCATMIN10 (VII) are linked to group I via eleven and 16 substitutions, respectively; they were isolated from single specimens of *C. minutus* in the USA.

#### *Leucocytozoon* spp. LTUR8

**Fig. 9:**
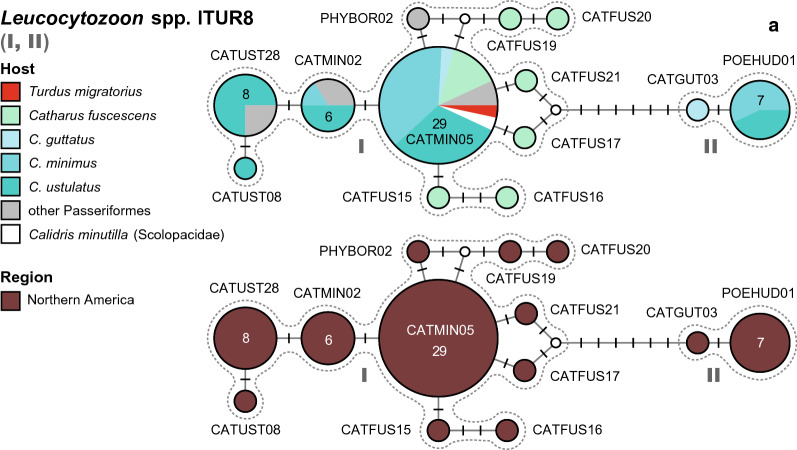
Median-Joining DNA haplotype network of partial (474 bp) *CytB* sequences of avian *Leucocytozoon* lineages belonging to clade lTUR8. The upper images show the host distributions and the lower image the geographic distributions. Groups of similar haplotypes potentially belonging to the same parasite species are framed in dotted lines and marked with Roman numbers in bold type

This network (Fig. 9) contains two groups of lineages from North America, connected via six substitutions. The first group (I) includes eleven lineages isolated from birds in North America: lCATMIN05 (29), lCATMIN02 (6), lCATUST28 (8), lCATUST08 (1), lCATFUS15 (1), lCATFUS16 (1), lCATFUS17 (1), lCATFUS19 (1), lCATFUS20 (1), lCATFUS21 (1), and lPHYBOR02 (1). lCATMIN05 was isolated from *C. minimus* (11), *C. ustulatus* (9), *C. fuscescens* (4), *C. guttatus* (1), *T. migratorius* (1), *Melospiza lincolnii* (Passerelidae, 1), *Melospiza georgiana* (Passerelidae, 1), and *Calidris minutilla* (Scolopacidae, 1) in the USA. lCATMIN02 was isolated from *C. ustulatus* (3), *C. minimus* (1), *Empidonax alnorum* (Tyrannidae, 1), and *Leiothlypis celata* (Parulidae, 1) in the USA. lCATUST28 was isolated from *C. ustulatus* (6), *Leiothlypis celata* (Parulidae, 1), and *Empidonax alnorum* (Tyrannidae, 1) in the USA. lCATUST08 was isolated from *C. ustulatus* (1) in the USA, and lPHYBOR02 from *Phylloscopus borealis* (Phylloscopidae, 1). Lineages lCATFUS15, lCATFUS16, lCATFUS17, lCATFUS19, lCATFUS20, and lCATFUS21 were isolated from single specimens of *C. fuscescens* in the USA. The second group (II) includes two lineages isolated from thrushes in the USA: lPOEHUD01 isolated from *C. minimus* (4) and *C. ustulatus* (3), and lCATGUT03 from *Catharus guttatus* (1).

#### Additional *Leucocytozoon* lineages from Turdidae birds

AFR187 was isolated from *Geokichla gurneyi* in Malawi, lASOT06 and lTUMER20 from *T. merula* in Austria, lCAP3 from *T. pilaris* in Sweden, lCATUST14 and lCATUST34 from *C. ustulatus* in the USA, lTFUS15 from *T. fuscater* in Colombia, lCOLBF21 from *H. mustelina* in the USA, lDUMCAR01 from *C. fuscescens* in the USA, lMETYR01 (= lTFUS11) from *T. fuscater* in Colombia, lMYAOCC01 from *M. occidentalis* in Mexico, lMYAUNI01 from *M. unicolor* in Honduras and Nicaragua, lMYAUNI03 from *M. unicolor* in Nicaragua, lTFUS14 from *T. fuscater* in Colombia, lTROAED02 and lMYARAL02 from *M. ralloides* in Peru, lTUMER03 from *T. merula* in Armenia and Portugal, lTUMER09 from *T. merula* in Lithuania, lTUMER10 and lTUMER18 from *T. merula* in Austria, lTUMIG12 and lTUMIG14 from *T. migratorius* in the USA, lTURALB04 from *T. albicollis* in Brazil, and lTUROLI06 from *T. olivaceofuscus* in Sao Tome and Principe. A phylogenetic tree with all *Leucocytozoon* lineages isolated from Turdidae hosts (and related lineages included in the networks) is provided in the Additional file 8. A summary of these *Leucocytozoon* lineages is provided in Additional file 10, which also includes information on the main host groups (bird families).

## Discussion

This study aimed to show the patterns of geographic and host distribution of haemosporidian lineages from birds of the family Turdidae based on *CytB* sequence data. Apart from providing a summary of the status quo regarding avian haemosporidian lineages of thrushes, this approach also facilitates the identification of lineages or groups of lineages, which potentially represent haemosporidian species not yet recognised. It should be noted that recent molecular studies combined with microscopic examination of haemosporidian parasites showed that morphologically readily distinguishable parasite species often show only negligible differences (a few base pairs) in the partial *CytB* sequences [46, 47]. Morphologically non-identified lineages are numerous in the networks, and some of them might represent distinct parasite species. In particular, there are some prominent haplotype groups, which likely represent still non-identified pathogen species and are worth primary attention in future taxonomic research, which this study can direct. Whereas the results section of this manuscript is organised according to the clades identified and lineages contained within, the authors of the present study aim to relate the sequence data with information available on morphologically described species in the discussion. In some cases, morphospecies described from Turdidae hosts were not yet identified by means of molecular genetics, and in others, the assignation of *CytB* lineages to certain morphospecies is questionable. Therefore, these issues are discuss based on data on the host and geographic distribution and vector competence studies.

It is also important to note that all currently used PCR-based protocols are often insensitive in reading co-infections of haemosporidians belonging to the same and different genera and subgenera, particularly in species of *Plasmodium* and *Haemoproteus* [48]. This problem remains insufficiently addressed in species of *Leucocytozoon*, but preferable amplification using certain primer sets certainly occurs [49]. This is also supported by the findings of [50], who tested metatranscriptomics as a tool to yield genomic data from avian haemosporidians. They showed that *CytB* sequencing greatly underestimates the total number of parasite lineages in mixed infections and samples with low parasitemia, particularly in *Leucocytozoon* infections. The transcriptomic approach detected 23% more haemosporidian infections in the sample studied than the standard DNA barcoding approach [50].

Moreover, some single reports of lineages normally completing their life cycles and producing gametocytes in birds of other families and orders might represent cases of abortive (or incomplete) development in thrushes. Abortive haemosporidian infections seem common in wildlife and might occur when vectors inject sporozoites in non-susceptible or partly susceptible (wrong) avian hosts [51]. Thereby a distinction has to be made between cases in which parasites undergo some rounds of replication but are unable to produce gametocytes, and others in which sporozoites are unable to infect the host cells and replicate at all. Such cases are dead-ends for transmission but might lead to positive results in PCR screenings. Unfortunately, many studies were not accompanied by microscopic examinations, and it remains unclear if some lineage reports in unusual avian hosts originate from competent infections resulting in the completion of the life cycle and the production of gametocytes, the essential stage for parasite transmission. In other words, the present data likely represent only a partial picture of host competence, but some formerly non-recognised patterns became visible and worth attention.

So far, only a few studies addressed species limits in avian haemosporidians. [52] compared sections of the mt *CytB* and the nc *DHFR*-*TS* gene in closely related lineages attributed to *Haemoproteus payevskyi* and *H. belobolskyi* and found that the two genes evolved in parallel, indicating that there is no or little exchange of genetic material between similar mt *CytB* lineages. They state that the presence of non-recombining parasite lineages within the same host species and geographic areas would indicate good species according to the biological species concept. This implies that most of mt *CytB* lineages could correspond to different biological species, whose number might approach 10,000 in the genera *Haemoproteus* and *Plasmodium* [52]. Nilsson et al. [45] followed a similar approach by studying four nc genes of closely related *CytB* lineages linked to *H. majoris* and found no recombination between nuclear genes of different lineages, concluding that all lineages might be considered reproductively isolated biological species. Similar results were also obtained in the first multi-gene approach on *Leucocytozoon* spp. by [16], providing evidence that reproductive isolation does occur between similar *CytB* lineages featuring indistinguishable morphotypes. In birds of the family Turdidae, the present study identified 82 lineages of *Plasmodium*, 37 of *Haemoproteus*, and 119 of *Leucocytozoon*, most of which are unique to this host group. The present *CytB* data alone does not allow determining the number of parasite species these lineages belong to, but given previous research, we may be dealing with a large number of reproductively isolated species of avian haemosporidians that infect thrushes. In terms of characterizing haemosporidian species, the authors of the present study also prefer a biological species concept as addressed by [52], and suggest that studying nc genes would greatly help to delimitate closely related parasite lineages. Nonetheless, the authors also think that it is of importance to relate the molecular genetic data to classical taxonomy, which is primarily based on the morphology of blood stages, and the vector and host susceptibility of the parasites. This issue is of high importance and was not addressed thoroughly enough in avian haemosporidian research. So far, more than 250 species haemosporidian species were described morphologically, but according to the MalAvi database (“Grand Lineage Summary Table”), less than half of these species (109) were characterised by means of molecular genetics. Moreover, *CytB* lineages were probably not linked correctly to morphospecies in some cases, which is partly addressed in the discussion below. Future research would greatly benefit if the molecular characterization of morphologically described species would be based on samples originating from the type host species and localities and, if available, include also type material of these parasites. The data on geographic and host distribution summarised here also might give directions for future taxonomic research by identifying groups of lineages, which might represent biologically isolated parasite species.

### *Plasmodium* parasites of the Turdidae birds

This study identified 82 *Plasmodium* lineages in thrushes, 58 of which clustered into the three clades shown as haplotype networks. Unlike in *Leucocytozoon* spp., most groups defined in the networks also include lineages isolated from other passeriform birds, which conforms to results of experimental observations showing broad specificity of many avian *Plasmodium* species [1, 53]. The diversity of *Plasmodium* lineages is higher in the genus *Turdus* compared to *Catharus*, and the networks pTUR1, pTUR2, and pTUR3 do not feature any lineage exclusive to *Catharus* spp. Only three lineages common in American *Turdus* spp. (pTUMIG03, pCATUST05, pCATUS06) were isolated from *C. ustulatus*, and only a single lineage (pCATUST21) was unique to the latter. One reason might be that birds of the latter genus are mainly distributed in the Americas, being less exposed to Old-World parasite lineages. Moreover, the number of *Turdus* species is considerably higher than that of *Catharus* with 80 compared to 12 species. The scarcity of *Plasmodium* spp. might also be explained by the observation that *Catharus* spp. mostly breed at high-latitude sites in North America where the overall prevalence of *Plasmodium* is quite low (Spencer Galen, personal communication). However, after this manuscript was submitted, [54] published a study on haemosporidian parasites of *Catharus* spp. sampled in eastern North America and found seven new lineages in two species of this genus. Two lineages isolated from *C. fuscescens* (pCATFUS10-11) differ by one and two bp from pCATUST05 (pTUR3, Fig. 2b). Four lineages isolated from *C. fuscescens* (pCATFUS12-14) and *Catharus bicknelli* (pCATBIC09) differ in one and three bp from pCATUST06 (pTUR2, Fig. 2a), respectively, and pCATBIC08 isolated from *C. bicknelli* by ten bp. This new study exemplifies that research in this field is still going on and that screening more bird species and individuals will render additional data.

The 58 Turdidae-specific *Plasmodium* lineages in the networks may be classified into 17 haplotype groups based on their genetic similarity and geographic and host distribution. Eight groups of similar lineages were found in New World Turdidae compared to nine groups in Old World Turdidae. Only four of the 17 groups of lineages were linked to the morphologically described species *P. vaughani* (pTUR1 I), *P. unalis* (pTUR1 IV), *P. matutinum* (pTUR2 I), and *P. lutzi* (pTUR2 II). In some cases, the assignation of *CytB* lineages to morphospecies might be incorrect. To address these issues, data from original species descriptions and further literature were reviewed and are discussed in the following.

#### Subgenus *Novyella*

*Plasmodium* (*Novyella*) *vaughani* was discovered and described from *T. migratorius* in Michigan, USA, where it was reported to be common in the type host [55]. The original description is fragmentary and difficult to use in taxonomy. However, this parasite was subsequently isolated from the type vertebrate host and examined in detail by [56], who re-described and validated this parasite species name, leaving no doubts about the morphology of its blood stages. Importantly, Manwell’s material from the type vertebrate host (*T. migratorius*) exists and was designated as neohapantotype of *P. vaughani* by [57]. Experimental studies showed that the American strain of *P. vaughani* does not complete its sporogonic development in *Cx. pipiens*, however, infection of canaries was possible [58]. Garnham [59] reported *P. vaughani* from other American thrushes, namely *T. leucomelas*, *T. rufiventris*, *T. anthracinus*, and *S. sialis*, but also from several other passeriform and non-passeriform birds.

Corradetti et al. [60] described the subspecies *Plasmodium* (*Novyella*) *vaughani merulae*, which was isolated from *T. merula* in Macerata, Macerata Province (Italy). Whereas the number of merozoites in the nominate form is often four (more rarely six and eight), *P. vaughani merulae* features a larger variation in this character, with on average six (more rarely four and eight) merozoites present in mature erythrocytic meronts. Contrary to the American strain, *P. vaughani merulae* completed its sporogonic development in *Cx. pipiens*, but infection of canaries was neither possible by blood inoculation nor by bites of infected mosquitos [60, 61]. The same results about non-susceptibility of canaries to blood-induced infections were obtained with European isolates of the *P. vaughani* lineage pSYAT5, including samples derived from *T. merula* [62]. Iezhova et al. [63] experimentally infected each one individual of *Erithacus rubecula* (Muscicapidae) and *Sylvia atricapilla* (Sylviidae) with *P. vaughani* isolated from another robin in Wilhelmshaven, Germany, and particularly noted the time differences between the prepatent periods of the German strain and the Italian strain studied by [61]. However, the length of the prepatent period markedly depends on the dose of infection and was impossible to compare in detail between these two strains [59].

Currently, *P. vaughani* is associated with the *CytB* lineage pSYAT05, which is common in *T. merula* throughout its entire distribution range, including New Zealand, to which the European blackbird was introduced [39, 40, 42]. Several studies recorded pSYAT05 in other European passerines (e.g., *Sylvia atricapilla, Alauda arvensis* and *Sturnus unicolor*), but the prevalences were much lower compared to *T. merula* (Data of some studies were not included because no sequences were published on GenBank; additional information can be found in the “hosts and sites table” of the MalAvi database). The European blackbirds screened for the present study showed a high prevalence for SYAT05, with 31% of individuals being infected. *Plasmodium vaughani* pSYAT05, was also the most common *Plasmodium*-lineage isolated from *Cx. pipiens* f. *pipiens* in Eastern Austria besides *P. matutinum* pLINN1 and *P. relictum* pSGS1 [64], but no proof was provided that sporozoites of *P. vaughani* (pSYAT05) developed in this mosquito. *Culex pipiens* might be the vector of pSYAT05, but the American *P. vaughani* strain does not complete sporogony in this *Culex* species according to [58], raising the question of whether pSYAT05 and the strain studied by [58] belong to the same *Plasmodium* species. Bearing in mind the available data, the subgeneric classification of these parasites (as *P. vaughani vaughani* and *P. vaughani merulae*) seems acceptable and worth using [1]. In North America, from where *P. vaughani* was described, pSYAT05 is extremely rare and was found only in *T. migratorius* in Michigan and Vermont [24, 65]. In a yet unpublished study, pSYAT05 was also found in *T. migratorius* in Pennsylvania and New York with 13 of 41 and 7 of 9 specimens being infected, respectively (Spencer Galen, personal communication). It remains to be investigated why pSYAT05 in *T. migratorius* was only found in the northeastern USA, despite that the distribution range of this host species includes large parts of North America. Another issue is that the insufficient original description of *P. vaughani* [55] potentially would also apply to *P. unalis*, a *Plasmodium* parasite recently described by [66] from *T. fuscater* in Colombia. Further malaria parasite research targeting *T. migratorius* in the northeast USA, combining microscopic and molecular diagnostic tools, could help to clarify the taxonomic issues outlined here.

*Plasmodium* (*Novyella*) *unalis* was described by [66] from *T. fuscater* in Bogotá, Colombia, where it was found in 13 specimens of the type host. Mantilla et al. [66] linked *P. unalis* to the MalAvi lineage pTFUS6 and mentioned several similar *CytB* lineages isolated from American thrushes and other birds in previous studies, pointing out that the parasite is probably widespread in the Americas. Tostes et al. [67] studied *Plasmodium* parasites of Brazilian thrushes and linked several additional lineages to *P. unalis*, namely pTURUF03, pTURUF04, and pTULEU06. The central haplotype of the *P. unalis* group (IV in Fig. 1) is lineage pTUMIG03, which was found in numerous Southern American thrushes as well as in *T. migratorius* and *C. ustulatus* in North America [14, 18, 24, 25]. Although the morphology of pTUMIG03 and other related lineages (e.g., pTUMIG23, pTURASS03, pTURFAL03, pTURFAL04, pTURALB02, pHYLMUS01, and pCOETOR01) has not been studied so far, they might belong to the same parasite species based on their genetic similarity. In contrast, pTULEU06, which was linked to *P. unalis* by [67], probably belongs to a separate yet undescribed species based on our analysis (group IV in Fig. 1). pTELEU06 and similar lineages (pTULEU01, pTULEU04, pTULEU05, pTULEU07, pTULEU08; group VII in Fig. 1), which were almost exclusively isolated from *T. leucomelas* in Brazil, differ from lineages of the *P. unalis* group (IV) in at least 12 bp (2.5%). Groups V (pTURALB03 and pTURALB05) and VI (pTURFLA01 and pTURFAL06), which are closely attached to the *P. unalis* group (IV), might belong to two other morphologically yet undescribed species of the subgenus *Novyella*. Moreover, some lineages of this group were found predominantly in one or two *Turdus* species only, potentially indicating the presence of host-specific cryptic species.

As mentioned above, it remains unclear why *P. vaughani* (pSYAT05) has been rarely reported in thrushes in the Americas and what parasite lineage [56], who re-described the species, was working with. Because *Novyella* parasites are diverse and morphologically similar, detailed analyses of their blood-stage characters are needed for species identification. The few morphological characters in the original description of *P. vaughani* [55] might also apply to *P. unalis* [66]. However, [66] compared blood stages of *P. unalis* pTFUS6 with those of type material of *P. vaughani* contained in the Garnham Collection at the Natural History Museum, London, and found several differences between the two parasites: both trophozoites and meronts of *P. vaughani* often feature two small distinct pigment granules and lack vacuoles, whereas those of *P. unalis* contain only a single large circular-shaped pigment granule and readily distinguishable vacuoles. These characters mentioned to be distinctive for *P. unalis* by [66] might look, at first glance, applicable also to *P. vaughani* as defined in the re-description by [56]. Manwell [56] noted that asexual blood stages of *P. vaughani* usually contain one large pigment granule, which is often accompanied by a second or even a third one, and he also noted that young trophozoites and early schizonts contain vacuoles. However, these species can distinguished based on the morphology of pigment granules in erythrocytic meronts. Predominantly, mature and nearly mature meronts of *P. unalis* contain a single, large pigment granule of regular, circular shape, which is not the case in mature meronts of *P. vaughani*, in which (1) one pigment granule is usually seen only in trophozoites and young developing meronts, but 2–3 pigment granules of different size are usually present in mature meronts and (2) if one granule is present in mature meronts, it is a clump of several granules and as a result of irregular (never of circular) shape. Additionally, mature and maturing erythrocytic meronts of *P. unalis* are mostly fan-like in shape but of irregular shape in *P. vaughani*. These features are worth being paid attention to during the morphological identification of these parasites.

*Plasmodium* (*Novyella*) *hexamerium* was described from the Eastern bluebird *Sialia sialis* in Illinois, USA, by [68], who also reported the parasite from *Geothlypis trichas* (Parulidae), *Dumetella carolinensis* (Mimidae), and *Zenaida macroura* (Columbidae). Manwell [69] summarised information on additional host species and noted that *P. hexamerium* is particularly common in North American sparrows. According to [1], the parasite was reported from more than 40 passeriform birds, but also from columbiform and strigiform birds, particularly in the Nearctic but also the Neotropical region. *Plasmodium hexamerium* is morphologically similar to *P. vaughani* but differs from the latter in the number of merozoites contained in erythrocytic meronts, which is usually six in *P. hexamerium* compared to four to six (sometimes eight) in the original American strain of *P. vaughani*. Both *P. hexamerium* and *P. vaughani* develop in canaries and do not complete sporogony in *Cx. pipiens* [1]. However, *P. hexamerium* develops in ducks and turkeys, which is not the case with *P. vaughani* [70], and developing erythrocytic meronts of *P. hexamerium* lack refractive globules. Although *P. hexamerium* was reported from various hosts in the Americas, it has not been linked to a *CytB* sequence yet.

*Plasmodium* (*Novyella*) *homopolare* was described recently from *Melospiza melodia* (Emberizidae) by [71] and linked to the lineage pBAEBIC02. The latter and genetically similar lineages were found in numerous North and South American birds, mainly in species of the Passeriformes (Parulidae, Emberizidae, Thraupidae, Trochilidae, Turdidae) but also Galliformes and Strigiformes [71–73]. Phylogenetically, *P. homopolare* BAEBIC02 and related lineages (Fig. 3) are closely related to the *P. vaughani*/*P. unalis* clade [74], with 4.1% mean difference (*p*-distance) in the *CytB* between the two clades. The general host distribution of *P. homopolare* is similar to that reported for *P. hexamerium* (Fig. 3, Additional file 1), and both species resemble each other in the morphological features of their blood stages. Minor differences concern the appearance of the cytoplasm in gametocytes, which is homogeneous in *P. hexamerium* and heterogeneous in *P. homopolare*, and the number of pigment granules contained in trophozoites (1–2 vs. 2–3) [1, 71]. Moreover, refractive globules are present in growing erythrocytic meronts of *P. homopolare* but absent in *P. hexamerium*. Based on this character, these parasites can be distinguished, but *P. homopolare* might have been overlooked or misidentified as *P. hexamerium* in some former studies due to the similarity of their blood stages. These parasites are probably closely related and more detailed examinations of blood-stages of *P. hexamerium* and comparison with those of *P. homopolare* are needed to clarify the taxonomic relationship between these pathogens. Morphological studies on additional lineages contained in the *P. homopolare* network (Fig. 3) might be helpful to clarify these issues.

*Plasmodium* (*Novyella*) sp. The analyses of *CytB* sequences revealed another group of four lineages (pTUR3, Fig. 2), which together form the sister clade to the *P. vaughani*/*P. unalis* clade (pTUR1, Fig. 1; Additional file 4). The *CytB* sequences of clades pTUR1 and pTUR3 differ in 5.4% (*p*-distance) from each other. The central haplotype pCATUST06 was isolated from few individuals of *C. ustulatus*, *C. aurantiirostris*, *M. ralloides*, *T. migratorius*, *T. leucops*, *T. serranus*, and *Grallaricula peruviana* (Grallaridae) in the Americas. Four of these species (*T. serranus*, *T. leucops*, *M. ralloides*, and *G. peruviana*) share similar distribution ranges in the Andes Mountains. The morphology of these *Plasmodium* lineages has not been assessed yet, but their close phylogenetic relationship to *P. vaughani* and *P. unalis* supports an affiliation to the subgenus *Novyella*.

### Subgenus *Haemamoeba*

*Plasmodium* (*Haemamoeba*) *matutinum*, Wolfson [75] found a *Plasmodium* parasite in *Hylocichla mustelina* from Baltimore, Maryland (USA), which she identified as a strain of *Plasmodium relictum* [formerly *Plasmodium praecox*]. Experimental infections of canaries showed that the parasite’s biology differed strongly from that of the nominate form of *P. relictum*, particularly in its strict periodicity and the high degree of synchronism in maturation (segmentation) of erythrocytic meronts [75]. The parasite strain was then isolated from *T. migratorius* in Kansas, Illinois (USA), and described as *Plasmodium relictum* var. *matutinum* by [76], who also confirmed previous findings in further infection experiments with canaries. Huff [76] also tested the susceptibility of *Cx. pipiens* for *Plasmodium relictum* var. *matutinum* and found it to be much lower than for previously tested American *P. relictum* strains (“Boston” and “Whitmore”). Infection rates for the latter two strains were almost 90% in experimentally fed *Cx. pipiens*, whereas only 2.5% mosquitos of a mixed culture of *Cx. pipiens* and *Cx. territans* were susceptible to *Plasmodium relictum* var. *matutinum* [76]. The main vector of the latter is probably *Cx. tarsalis*, which in experimental studies showed infection rates of up to 84% [77].

Corradetti et al. [78] identified a European strain of *P. relictum* var. *matutinum* in the redwing *Turdus iliacus* in Italy and raised the parasite to species rank. They showed that *Cx. pipiens* easily could be infected with the European strain, unlike with the North American one [78, 79]. Another difference is the much higher virulence reported for the Italian strain in canaries. Inoculation with the Italian strain was fatal for the canaries and led to death after five to 20 days [78]. However, in canaries experimentally infected with the North American strains, infection built up rapidly for 4 to 5 days but then declined into latency [59]. Nonetheless, susceptibility to mosquito species hardly can be considered as a good taxonomic character because it can be readily changed by selection [1, 59].

Valkiūnas et al. [80] isolated pLINN1 from *Luscinia luscinia* (Muscicapidae) in Lithuania and linked it to *P. matutinum* because the morphological characters of the blood stages of pLINN1 corresponded well with those in the parasite’s original description. Experimental inoculations with infected blood showed that domestic canaries are susceptible to pLINN1, but the parasitemia was low and the infection was not lethal, similar to observations on the North American *P. matutinum* strain [80]. However, only two canaries were exposed, and estimating the virulence is difficult based on the available data. *Culex pipiens* f. *pipiens* might be a vector of pLINN1, which was reported in this mosquito, but sporogonic stages were not studied and it remains unclear if the sporogony completes and sporozoites develop in this mosquito species [64, 81]. pLINN1 was found also in 30% of *T. merula* and 22% of *T. philomelos* specimens investigated in the present study. The lineage pAFTRU5, which, based on genetic similarity, probably also belongs to the same parasite species, was found in 1.6% of *T. merula* specimens. Further records in *T. merula* from Portugal, Western Russia, Armenia, and Morocco [8] indicate that pLINN1 and pAFTRU5 are co-occurring with their avian host species throughout their whole distribution ranges. The data of these studies indicate that *P. matutinum* pLINN1 and pAFTRU5 are the most common *Plasmodium*-lineages of *T. merula* apart from *P. vaughani* SYAT05. The parasite lineages were also introduced to New Zealand, probably together with their host species [42]. In North America, pLINN1 and pAFTRU05 were recorded only in single specimens of *T. migratorius* in Michigan [24] and Nebraska [82], respectively. pLINN1 was also detected in *Cx. pipiens* and *Cx. restuans* in Ithaca, New York [83].

Based on the present data, *T. merula* is the main vertebrate host of the lineage pLINN1. This parasite lineage is present but rare in North America, which is the type locality of *P. matutinum*. Considering the available data about differences in (1) the host and geographic distribution of molecular genetic lineages, (2) the occurrence and susceptibility in mosquitoes, (3) the periodicity and degree of synchronism in maturation (segmentation) of erythrocytic meronts, and (4) the virulence of different isolates in experimentally infected canaries, pLINN1 and related European strains probably do not correspond to the parasite originally described in *T. migratorius* as *P. relictum* var. *matutinum*. pLINN1 probably differs also from the Italian strain studied by [79], because the latter was lethal to canaries. Further experimental studies are needed to test these hypotheses.

*Plasmodium* (*Haemamoeba*) *giovannolai* was described from *T. merula* in Lazio, Italy, by [84], who reported the parasite only from the type host. Canaries were susceptible and died within a month after experimental infection, and also *Cx. pipiens* easily could be infected [85]. In biology and morphology, *P. giovannolai* is remarkably similar to the European strain of *P. matutinum* depicted in [1], which might be the same strain later linked to pLINN1 and isolated from *Luscinia luscinia* [80]. However, the pLINN1 isolate from *L. luscinia* was not lethal in canaries, and is different in this respect from *P. giovannolai*. Valkiūnas [1] suggests that *P. giovannolai* might be a subspecies of *P. matutinum*. Additional support for a close relationship between the two species comes from sequence data of haemosporidian parasites because all *Haemamoeba* lineages isolated from *T. merula* (pLINN1, pAFTRU5, pTUMER06, and pTUMER12) exclusively grouped into a single sequence cluster (pTUR2, Fig. 2). Therefore, it might be possible that the original *P. giovannolai* represents one of the lineages currently considered belonging to *P. matutinum*. However, despite morphological similarities, the two strains differ notably in one feature. Phanerozoites of *P. giovannolai* develop almost exclusively in the spleen of canaries but were never seen in the brain, whereas those of *P. matutinum* are found in numerous organs [1]. A comprehensive study on haemosporidioses of *T. merula* and *T. philomelos* confirmed that phanerozoites of *P. matutinum* pLINN1 develop in multiple organs [86], but tissue stages of pLINN1 were not yet studied in canaries. Additional experimental infections of canaries with pLINN1 could help confirm differences between *P. giovannolai* and *P. matutinum* pLINN1 regarding the location of tissue stage formation. It is worth mentioning that the same *Plasmodium* strain might be markedly different in the ability to produce phanerozoites in the brain of different avian host species [87], so this feature should be used carefully in taxonomic research.

*Plasmodium* (*Haemamoeba*) *lutzi* was described by [88] from *Aramides cajaneus* (Rallidae, Gruiformes) in São Paulo, Brazil. Gabaldon and Ulloa[89] report the parasite also from birds in Venezuela and suggest that records made by [90] from Columbian birds refer to the same morphospecies. The *CytB* lineage pTFUS05, isolated from two individuals of *T. fuscater* in Bogotá, Colombia, was linked to *P. lutzi* because of morphological similarities [13]. Renjifo et al. [91] linked several additional lineages to *P. lutzi*, namely pDIGLAF01, pDIGLAF02, pDIGCYA08, and pCATUST05 [original lineage names differ in the publication]. pTUMIG22, isolated from *T. migratorius* in the USA and *T. plumbeus* in the Caribbean [22], is also grouped with the latter lineages in clade pTUR2 (Fig. 2). Of these, pCATUST05 is the most common lineage, having been found in several species of American thrushes, birds of other passeriform families, and in the owl species *Aegolius acadicus* [e.g., 18, 19, 92]. Concerning the identification of pTFUS05 as *P. lutzi*, there are some taxonomic issues, which deserve attention. The type host of *P. lutzi* and the host of pTFUS05 belong to separate bird orders, the Gruiformes, and the Passeriformes. Moreover, the type localities São Paulo (Brazil) and Bogotá (Colombia) are more than 4000 km apart from each other and situated in altitudinal zones differing in 2000 meters. [13] also report morphological differences between pTFUS05, the sample studied by [89], and the original *P. lutzi*. For instance, the original strain of *P. lutzi* differs from pTFUS05 in various size measurements and particularly in the number of merozoites developed in erythrocytic meronts (16–28 vs. 6–26) [13]. Based on the data summarised in the present study, pTFUS05 probably does not belong to the original strain of *P. lutzi*. Further studies on *Plasmodium* parasites of the type host, *A. cajaneus*, at sites located closer to the type locality would be required to confirm or refute the identity of pTFUS05 as *P. lutzi*. So far, only a single *Plasmodium* lineage (pARACAJ01) has been isolated from a specimen of *A. cajaneus* kept in the São Paulo Zoo [93]. pARACAJ01 differs in about 5% (*p*-distance) from its closest related *Plasmodium* lineages and in more than 8% from pTFUS05. Morphological identification of the latter lineage was not possible, because only young stages, mainly meronts, were present in the blood of this specimen (personal communication, Carolina R. F. Chagas).

*Plasmodium* (*Giovannolaia*) *circumflexum* was described by [94] from *Turdus pilaris* in Germany. This morphospecies was then reported from over 100 bird species belonging to the orders Passeriformes, Anseriformes, Columbiformes, Coraciiformes, Charadriiformes, Falconiformes, Strigiformes, and Galliformes [1]. Valkiūnas et al. [95] linked *P. circumflexum* to pSW5, a lineage isolated from birds of the Passeriformes, Anseriformes, Gruiformes, and others, but not from Turdidae. They also mention the lineage pTURDUS1 to be closely related to pSW5. pTURDUS1 and pBT7, differing only in one substitution from each other in the *CytB* barcode section, show similar host distributions as reported for *P. circumflexum* previously [1] but they differ in 2.3% (*p*-distance) from pSW5. pTURDUS1 and pBT7 were recorded mainly in passeriform birds, including some Turdidae species (pTURDUS1: *T. merula* and *T. philomelos*; pBT7: *T. pilaris* and *T. migratorius*), but also in birds of the orders Accipitriformes, Anseriformes, Strigiformes, and Charadriiformes (see MalAvi database). At least pTURDUS1 was also considered a lineage of *P. circumflexum* by [96] and [62]. This pattern might indicate the presence of cryptic species.

*Plasmodium* (syn. *Proteosoma*) *tumbayaensis* was briefly described by Mazza and Fiora [97] from *Planesticus anthracinus*, which probably is a synonym of *Turdus chiguango*, in Tumbaya (Argentina). Blood stages of this parasite are similar to *P. vaughani*, to which it was synonymised by [98]. Due to the marked genetic diversity of *Novyella* parasites in Neotropics, validation of this species name is probably possible, but additional research on its biology is needed.

### *Haemoproteus* parasites of the Turdidae birds

The diversity of *Haemoproteus* lineages in thrushes is comparably low. In total, 37 *Haemoproteus* lineages were identified in thrushes, 17 of which clustered in the two networks hTUR1 (Fig. 4) and hTUR2 (Fig. 5). The 17 lineages in the networks can be classified into at least four or five groups belonging to distinct *Haemoproteus* species. North American thrushes harbor a relatively high diversity of *Haemoproteus* lineages, with nine lineages in the *H. minutus* group (Fig. 4), four lineages in the *H. majoris* group (Fig. 5), and about 20 more rare lineages (mainly from *C. ustulatus* and South American thrushes) in different clades (Additional file 6). Both the *H. minutus* and *H. majoris* clades contain multiple lineages, which were isolated from and complete development with production of gametocytes in non-Turdidae hosts, indicating either relatively recent host switches between Turdidae and other passeriform birds or possible high susceptibility of thrushes to infections, which came from other bird species but abort their development. From this point of view, birds of this group and their *Haemoproteus* parasites seem to be good model organisms to address abortive development in haemoproteids.

Bennett [99] described *Haemoproteus* (*Parahaemoproteus*) *fallisi* from *T. migratorius* in Newfoundland, Canada. At the type locality, they found the parasite in 28% of sampled *T. migratorius*, 22% of *Catharus minimus*, and 1% of *C. ustulatus*. Bennett [99] speculated that the same parasite strain might have been recorded in numerous species of Turdidae and Muscicapidae worldwide. Considering that each about 200 specimens of *T. migratorius* and *C. minimus* were screened for haemosporidians in recent molecular genetic studies (see MalAvi database), the corresponding *CytB* lineage should have been detected already. *Haemoproteus* lineages isolated from *T. migratorius* and *C. minimus* cluster in either the *H. minutus* lineage group (Fig. 4) or the *H. majoris* group (Fig. 5). In particular, *H. majoris* shares several morphological characters with *H. fallisi*: the advanced growing gametocytes both adhere to the erythrocyte nuclei and envelope and slightly displace the nuclei laterally; gametocytes grow around the erythrocyte nucleus, but do not encircle it completely; young gametocytes of *H. fallisi* are frequently amoeboid, which also applies to *H. majoris*; gametocytes of *H. fallisi* contain in average 15 (11–21) and those of *H. majoris* about 10 pigment granules, which usually are randomly scattered throughout the cytoplasm. However, fully-grown gametocytes of *H. fallisi* are smaller in length and usually do not reach poles of erythrocytes, a character not characteristic in fully-grown gametocytes of *H. majoris*. The original *H. fallisi* strain might, therefore, correspond to one of the North American *CytB* lineages clustering in the *H. majoris* group, whose morphological traits should be similar to the original description of *H. fallisi* and need additional research on the avian type host *T. migratorius*. To clarify the relationship between *H. fallisi* and other *Haemoproteus* species, morphological studies of *Haemoproteus* parasites of *T. migratorius* and *C. minimus* in the type locality (Newfoundland) are suggested. This study also shows that *H. minutus* is present in *T. migratorius* and *C. minimus* but has not been documented in these birds on gametocyte stage level, which needs clarification.

*Haemoproteus* (*Parahaemoproteus*) *majoris* was described by [100] from *Parus major* (Paridae) in Metz (France). The parasite was reported from more than 80 passeriform species in the Holarctic, Ethiopian, and Oriental regions [1]. Most host species belong to the families Paridae, Sylviidae, Muscicapidae, and Fringillidae, but Turdidae were not mentioned as vertebrate hosts of *H. majoris*. However, four lineages (hPHYBOR04, hPOEATR01, hTUMIG08, and hTUMIG21), isolated from the North American thrushes *T. migratorius*, *C. minimus*, and *C. guttatus*, differ only in few bp from lineages currently attributed to *H. majoris*. All other lineages in the *H. majoris* group (Fig. 5) were isolated from non-Turdidae passeriform birds in Western Europe, Eastern Europe, and Africa. There is only a single record of hPARUS1 from *T. merula* in Armenia [8], which might be a case of an abortive infection. Nilsson et al. [45] linked several lineages to *H. majoris* (hPARUS1, hPHSIB1, hWW2, hCCF5, and hCWT4) and discussed that these might belong to a complex of cryptic species. The North American lineages differ in one to a few bp from those linked to *H. majoris* and should be considered as part of the same species group. Detailed morphological studies of these lineages are required to support the latter assumption.

Cleland and Johnston [101] described *Haemoproteus* (*Parahaemoproteus*) *geocichlae* from *Zoothera lunulata* in New South Wales, Australia. The parasite has not been reported from other host species and sampling sites since. Beadell et al. [7] screened samples of 105 bird species from Australia and Papua New Guinea and detected hZOOLUN01 in one of two *Z. lunulata* specimens. hZOOLUN01 differs from *H. minutus* hCOLL2 in one substitution and is the only *Haemoproteus* lineage isolated from *Z. lunulata* so far. Beadell et al. [7] mention that they prepared blood films from this bird but did not publish pictures of the parasites. To clarify the identity of this parasite lineage, morphological studies of gametocytes in *Z. lunulata* in Australia and the designation of type material are required.

The *Haemoproteus minutus* group includes several parasite species, which are common in thrushes: *H. minutus*, *H. pallidus*, and *H. homominutus*. Interestingly, the *CytB* lineages of these species differ only in a few bp from each other but show different host preferences and distinct morphological features both at gametocyte and sporogony stages. Most lineages of these species have Holarctic distributions, but some lineages of this species group were found also in Australia (GERPAL02, PARPUN01, SERCIT02, and ZOOLUN01), sub-Saharan Africa (AFR076, AFR081, BERMAD02, ILCLE01, TUROLI05, XANZOS01, and XANZOS02), and South America (RAMCAR06) (Fig. 4).

*Haemoproteus* (*Parahaemoproteus*) *minutus* was described by [102] from *T. merula* sampled at the Curonian Spit in Kaliningrad District, Russia. Palinauskas et al. [44] linked hTURDUS2, hTUCHR01, and hTUPHI01 to *H. minutus* because of their genetic similarity and common morphological traits. Differences in host composition indicate that hTUPHI01 might belong to a separate, cryptic species. Whereas hTURDUS2 was mostly isolated from *T. merula*, hTUPHI01 was almost exclusively isolated from *T. philomelos* (Fig. 4). Although the distribution ranges of *T. merula* and *T. philomelos* vastly overlap in Europe and Western Asia, the two bird species are not closely related and therefore might be prone to infection with different parasites. Moreover, the lineages hTUPHI01 and hTUCHR01/hTURDUS2 are not directly connected in the network, but via the intermediate haplotype hCOLL2, which belongs to a morphologically distinct parasite species, *H. pallidus*. hTUCHR01 also differs from hTURDUS2 in geographic and host distribution, being more common in Russia and Asia.

*Haemoproteus* (*Parahaemoproteus*) *homominutus* was recently described from *T. viscivorus* in Lithuania by [46], who reported the parasite also from *T. merula*. The Lineage hCUKI1 was so far only isolated from the type host *T. viscivorus* in Lithuania and Morocco, and *Culicoides circumscriptus* in Turkey. The records from *C. circumscriptus* were published in GenBank (KX831074 und MF095643) only, and the vector competence for *H. homominutus* has not been tested yet. The lineage hCUKI1 has not been isolated from other Turdidae species and might preferably infect *T. viscivorus*, although it was also seen in *T. merula* on gametocyte stage [46].

Valkiunas and Iezhova [103] described *Haemoproteus* (*Parahaemoproteus*) *pallidus* from *Ficedula hypoleuca* (Muscicapidae) in Kaliningrad District, Russia. According to [1], the distribution of *H. pallidus* is insufficiently investigated but includes the Palearctic and the Ethiopian zoogeographical regions. [104] linked the *CytB* lineages hCOLL2 and hPFC1 to *H. pallidus*. The main hosts of hCOLL2 and hPFC1 are the two Muscicapidae species *Ficedula albicollis* and *F. hypoleuca* with more than 300 individual records of each lineage, whereby hPFC1 is more common in *F. hypoleuca* and hCOLL2 in *F. albicollis* [105–107] (sequences of the latter studies were not published on GenBank and therefore not included in the DNA haplotype network in Fig. 4). Both Muscicapidae species are long-distance migrants with breeding habitats in Europe and Western Asia and wintering habitats in Sub-Saharan Africa. In Cameroon and Gabon, hCOLL2 was also found in the white-tailed ant thrush *Neocossyphus poensis* [108], but there is only a single record from *T. merula* in Western Russia [8]. However, in Northern and Central America hCOLL2 was almost exclusively isolated from thrushes, namely *T. migratorius*, *T. assimilis*, *C. minimus*, and *C. ustulatus*. Concluding, *H. pallidus* is not only distributed in the Palearctic and the Ethiopian regions, but also the Nearctic. Interestingly, there are still no reports about the presence of gametocytes of *H. pallidus* in American thrushes. Nevertheless, microscopical blood slide analyses are required to confirm that North American thrushes are competent hosts for hCOLL2 and related lineages and that the parasites develop gametocytes in thrushes. The lineages hCATUST22, hTURASS04, hTUMIG07, hTUROLI05, and hZOOLUN01, which are linked to hCOLL2 via one or two substitutions, were also isolated from thrushes, but the morphological identification of these parasites has not been developed yet.

### *Leucocytozoon* parasites of the Turdidae birds

Regarding the diversity of *CytB* lineages, *Leucocytozoon* represents by far the most diverse genus of haemosporidians in thrushes with 119 lineages, 94 of which clustered into the eight clades shown as haplotype networks. Most of these lineages were exclusively isolated from birds of the family Turdidae and therefore seem to be specifically adapted to this host group. Moreover, unlike *Plasmodium* and *Haemoproteus* parasites, the *Leucocytozoon* lineages are almost exclusively restricted to either species of the genus *Turdus* or *Catharus*, but they are usually not shared between them. The clades lTUR2 (Fig. 6b), lTUR3 (Fig. 7a), and lTUR4 (Fig. 7b) contain almost exclusively lineages isolated from *Turdus* spp., and lTUR5 (Fig. 7c), lTUR6 (Fig. 8a), and lTUR7 (Fig. 8b) from *Catharus* spp. Only clade lTUR1 (Fig. 6a) contains *Leucocytozoon* lineages isolated from species of both bird genera, however, in distant subclades.

The 94 Turdidae-specific *Leucocytozoon* lineages in the networks may be classified into at least 38 groups, which potentially belong to distinct species, but only a few have been linked to morphospecies yet. [16] identified four parasite lineages in thrushes (lCATGUT02, lTUMIG15, and lTUMIG11), which matched *L. majoris* morphotypes, and one lineage (lCATMNI01), which matched *L. dubreuili* morphotypes (Fig. 1 and Additional file 2 in [16]). However, the three *L. majoris*-like lineages and several additional ones isolated from other bird families (hACAFL01, hBT1, hCB1, hJUNHYE04, and hPHYBOR1) are genetically distinct and were mostly classified as separate species in the multi-gene species delimitation analysis of [16]. These lineages might represent cryptic species sharing similar morphological traits.

The majority of these *Leucocytozoon* lineage groups (25) was isolated exclusively from thrushes in the Americas, compared to eleven from Old World *Turdus* species. Only two groups (lTUR1 II and lTUR3 I) contain lineages from both New-World and Old-World *Turdus* species. The diversity was particularly high in North American thrushes. In South America, *Leucocytozoon* spp. were only isolated from thrushes native to the Andes Mountains (*T. fuscater*, *T. falcklandii*, *T. serranus*, *T. nigriceps*, *T. chiguanco*, and *C. fuscater*). This is in accord with literature reporting *Leucocytozoon* to be generally rare in the Neotropical region, which might be related to the absence of suitable vectors in lowland areas of South America [1]. Simuliidae black flies are present in South American tropical lowland areas [e.g., 109, 110], but the local species might not be susceptible to *Leucocytozoon* spp. [111] summarised records of *Leucocytozoon* spp. in South America and also present a case of *Leucocytozoon* infecting a host population in Amazonia. They suggest that high average temperatures may be constraining the diversity of *Leucocytozoon* in lowland tropical South America, because parasites of this genus generally require lower temperatures for the development in their blackfly vectors [111]. A general problem in the classification and identification of *Leucocytozoon* species is the low number of distinctive morphological characters in their blood stages. Gametocytes of *Leucocytozoon* parasites do not produce pigment granules, whose number, morphology, and location are important morphological traits for *Plasmodium* and *Haemoproteus* species. Unlike *Plasmodium* parasites, *Leucocytozoon* species do not multiply in the blood and do not produce blood-cell meronts, which provide additional morphological features for malaria parasite classification. Consequently, the main criteria for delimiting *Leucocytozoon* species have been the shape and location of infected host cell nuclei and the morphology of host cell-parasite complexes (roundish or elongate, with different shapes of the cytoplasmic processors in the latter). Additionally, the occurrence in different host groups was also taken into consideration during *Leucocytozoon* species description, assuming that host switches between different bird families and orders represent unlikely or rare events. Therefore, morphologically similar parasites isolated from birds of separate orders or families were often considered distinct species [1, 53]. These morphological characters certainly have taxonomical value, and many readily distinguishable *Leucocytozoon* species exist and were described, but the marked overlap of main diagnostic features in different *Leucocytozoon* species is also evident. This study supports the existence of cryptic speciation in the genus *Leucocytozoon*. This is not surprising due to (1) the poor morphological differentiation of leucocytozoid blood stages being readily accessible for research, and (2) because of the lack of data about other stages of development for the majority of described species. It is probable that many more *Leucocytozoon* species exist, but they hardly can be distinguished solely based on the blood stages of the parasites. Examination of vectors and parasite sporogonic and tissue stages might be needed to morphologically characterise these species. The difficulties in morphological identification may explain, why only a few *Leucocytozoon* species were described in the last decades, and only little progress was made regarding linking *CytB* lineages to morphospecies.

To go ahead with the molecular characterization of *Leucocytozoon* parasites, the identification of *CytB* lineages of the most common species parasitizing passerines (*Leucocytozoon fringillinarum*, *L. majoris*, *L. dubreuili*, and others) are needed. These parasite species names have taxonomic priority due to their early dates of description [112], but their lineages remain unidentified from the type hosts and type localities. The lack of this information precludes further taxonomic work on leucocytozoids in passeriform birds. The main obstacle of linking parasite morphotypes with their lineages is that co-infections are common and even predominate, being visible in blood films but not always detectable or distinguishable using standard PCR-protocols [49]. In the case of *Leucocytozoon* parasites this problem seems to be even more severe than in the case of *Plasmodium* and *Haemoproteus* infections [48], but still remains insufficiently addressed. After this manuscript was submitted, [54] published a study for which they screened 460 individuals of *C. minimus*, *C. fuscescens*, and *Catharus bicknelli* for haemosporidian parasites. They identified 16 new *Leucocytozoon* lineages (lCATBIC01–06, lCATBIC10–13, lCATFUS01–02, lCATFUS04–09, and lCATMIN11) in these three bird species, which were not included in the present study. All of these lineages would cluster in the *Leucocytozoon* networks presented here, differing from already published lineages by one to six bp. This study exemplifies that this area of research is still developing and that sampling of larger numbers of individuals and additional host species will refine our understanding on haemosporidians of thrushes.

*Leucocytozoon dubreuili* was briefly described by [113] from *Turdus* sp. in Tonkin, Vietnam. The French name of the type host is “grive”, which translates to “thrush”. More than 15 Turdidae species are known from Vietnam, half of which include “grive” in their French name (Birds of Vietnam; http://sibagu.com/vietnam/turdidae.html), wherefore the type host cannot be deduced from the original description. Later, the parasite was reported from over 60 species of passeriform birds from the Holarctic, Ethiopian, and Oriental regions, but there are only a few records from the Neotropical and Australian regions. Moreover, sporogonic development of *L. dubreuili* was reported in several Simuliidae species belonging to the three different genera *Cnephia*, *Prosimulium*, and *Simulium* [1]. As long as the vertebrate type host is unknown, linking the species to a *CytB* lineage would be afflicted with severe uncertainties. Although morphological studies of haemosporidians of South-East Asian and Southern Asian birds are available (e.g., [114]), these regions still are black spots in regard to diversity of *CytB* lineages. These regions harbor a large variety of Turdidae species from different genera, but almost no sequence data of their haemosporidian parasites are available. Moreover, it seems unlikely that records of *L. dubreuili* from non-Turdidae passeriform birds refer to the same parasite species because *Leucocytozoon* lineages of Turdidae usually seem to be restricted to members of the same family or species (data of the present study). Two other *Leucocytozoon* species were also described from Turdidae hosts but were later synonymised with *L. dubreuili* based on the morphological identity of their blood stages and host cells by [115]. *Leucocytozoon giovannolai* was described from *T. iliacus* in Northern Africa by [116], and *Leucocytozoon mirandae* from *T. merula* in Portugal by [117]. The latter two taxa were isolated from different host species and geographic regions, and therefore might be distinct from the parasite strain originally described as *L. dubreuili*. These species names might be validated if blood samples from type hosts and their *CytB* sequences are available.

*Leucocytozoon maccluri* was described by [118] from *Zoothera marginata* in Thailand. The parasite was found in two specimens of the type host but was never reported from other host species. The morphology of the gametocytes and their host cells is unique and differs strongly from that of other *Leucocytozoon* species. Unfortunately, haemosporidian sequences of *Z. marginata* were not published so far.

### Availability of data: GenBank vs. MalAvi database

The MalAvi database was established by [10]. It first promoted the use of a 478 bp section of the mt *CytB* as a standard DNA barcode sequence and introduced a unified naming scheme for avian haemosporidian lineages. Moreover, it summarises information on geographic and host distribution of *CytB* lineages and provides references to the original publications and parasite descriptions, including details on their morphological characters [10]. Importantly, the sequences of new lineages deposited in MalAvi database are checked for their quality and are minimised in regard to possible misidentifications, which remain an insufficiently controlled problem in GenBank [119]. However, a sensitive problem for future research concerning MalAvi database is that authors of many publications made the data only available in the MalAvi database, but did not submit the sequences to GenBank or other government-financed sequence repositories. The only *CytB* sequences contained in MalAvi database are those in the “Grand Lineage Summary” table, which features a single sequence for every unique lineage, but the “Host and Sites” table, which summarises information on host species, localities, and references, contains lineage names only. Unless the sequences were not submitted in parallel to GenBank or other platforms, information on sequence length and quality is lost. Moreover, these records will not show up in GenBank searches, which is disadvantageous for future research, and which was also a problem for the data analysis of the present study. To ensure that records stay permanent, it is recommended to submit sequences of all individual samples (including information on host species, geographic origin, age, etc.) to GenBank. Alternatively, it is possible to submit one sequence per lineage detected to GenBank and provide data for individual host specimens either in the publication or as supporting information (e.g., [8]). This would allow retrieving the data with GenBank searches and at the same time would facilitate incorporation into the MalAvi database. The GenBank submission process currently also involves sequence checks, which detect interruptions of the coding frame and other errors, which helped to improve the quality of sequences in more recent GenBank entries. Concluding, the authors of the present study strongly encourage researchers to deposit their highly valuable data to GenBank in all cases. This will open opportunities to more precise research of the sort presented in this study as well as enrich the data available in MalAvi.

## Conclusion

This study represents the first attempt to summarise the geographic and host data on haemosporidian parasite lineages of a whole bird family, the Turdidae. Based on the available information from *CytB* sequences, the present study identified 82 *Plasmodium*, 37 *Haemoproteus*, and 119 *Leucocytozoon* lineages in thrushes, of which 68, 28, and 112 lineages, respectively, were mostly found in this host group. The DNA haplotype network analyses allowed identifying numerous clearly distinct groups of lineages, which have not been linked to morphospecies yet, and many of them likely belong to yet undescribed parasite species. This requires particular attention from taxonomists. The analyses also revealed several cases in which *CytB* lineages probably were assigned to the wrong morphospecies. These taxonomic issues are addressed in detail by comparing the host and geographic distribution of the *CytB* lineages with data from the original species descriptions and further literature. Recent molecular studies have recognised a huge genetic diversity of avian haemosporidian parasites, but there is still no methodology suggested how to use this information in practical work on parasite taxonomy and classification, which is essential for better understanding pathogen biodiversity. This study shows directions for this work by identifying groups of closely related lineages and evaluating their geographic and host distribution. MalAvi database remains a reliable and necessary source for molecular genetic studies of avian haemosporidians, which summarises the information on all parasite lineages and related literature. However, the authors also discuss obstacles related to the availability of sequence data for similar analyses and emphasise that all sequence data should be deposited in GenBank as well.

## Supplementary information

**Additional file 1.** Main table containing information (GenBank accession numbers, MalAvi lineage names, DNA sequences, host names, country names, and references) on the sequences used in the present study.

**Additional file 2.** *Plasmodium* lineages found in Turdidae birds (haplotype networks).

**Additional file 3.** Additional *Plasmodium* lineages found in Turdidae birds.

**Additional file 4.** Maximum likelihood tree of *Plasmodium CytB* lineages (474 bp) included in the present study. The total geographic distribution of lineages according to the United Geo-scheme is based on data from the MalAvi data base.

**Additional file 5.** *Haemoproteus* lineages found in Turdidae birds (haplotype networks).

**Additional file 6.** Additional *Haemoproteus* lineages found in Turdidae birds.

**Additional file 7.** Maximum likelihood tree of *Haemoproteus CytB* lineages (474 bp) included in the present study. The total geographic distribution of lineages according to the United Geo-scheme is based on data from the MalAvi data base.

**Additional file 8.** Maximum likelihood tree of *Leucocytozoon CytB* lineages (474 bp) included in the present study. The total geographic distribution of lineages according to the United Geo-scheme is based on data from the MalAvi data base.

**Additional file 9.** *Leucocytozoon* lineages found in Turdidae birds (haplotype networks).

**Additional file 10.** Additional *Leucocytozoon* lineages found in Turdidae birds.

## Data Availability

All sequences and related data are contained in Additional file 1. The sequences generated for the present study were deposited in NCBI GenBank under the accession numbers MT912098–MT912353.

## Abbreviations

BI: Bayesian inference
bp: Base pair/s
*CytB*: *Cytochrome b*
*COI*: *Cytochrome c oxidase subunit I*
ML: Maximum likelihood
mt: Mitochondrial
nc: Nuclear
AUS: Australia
CAF: Central Africa
CAM: Central America
CAR: Caribbean
EAF: Eastern Africa
EAS: Eastern Asia
EEU: Eastern Europe
NAF: Northern Africa
NAM: North America
SAF: Southern Africa
SAM: South America
SAS: Southern Asia
WAF: Western Africa
WAS: Western Asia
WEU: Western Europe
PAC: Pacific
