# Supplementary material for: Geographic and host distribution of haemosporidian parasite lineages from birds of the family Turdidae
Source: Malar J. 2020 Sep 15;19:335. doi: 10.1186/s12936-020-03408-0 (PMC7491118; doi:10.1186/s12936-020-03408-0)
Supplement: Supplementary file 2 — Additional file 2. Plasmodium lineages found in Turdidae birds (haplotype networks). [file 12936_2020_3408_MOESM2_ESM.docx]

**Additional file 2: *Plasmodium* lineages found in Turdidae birds (haplotype networks)**

| **Clade** | **group** | **Species** | **Turdidae-specific** | **Lineage** | **Main host groups (family, species)** | **Distribution** |
| --- | --- | --- | --- | --- | --- | --- |
| pTUR1 | I | *P. vaughani* | 1 | pSYAT05 | Turdidae (*T. merula, T. migratorius, T. philomelos, T. viscivorus*), Sylviidae, etc. | WEU, EEU, WAS, NAF, NAM, EAS |
|  |  | *P.* cf. *vaughani* | 1 | pAFTRU08 | Turdidae (*T. pelios*) | CAF |
|  |  | *P.* cf. *vaughani* |  | pDONAN08 | *Aedes (O.) caspius* | WEU |
|  |  | *P.* cf. *vaughani* | 1 | pTUMER04 | Turdidae (*T. merula*) | WEU |
|  |  | *P.* cf. *vaughani* | 1 | pTUMER05 | Turdidae (*T. merula*) | WEU |
|  |  | *P.* cf. *vaughani* | 1 | pTUMER13 | Turdidae (*T. merula*) | WEU |
|  |  | *P.* cf. *vaughani* | 1 | pTUMER14 | Turdidae (*T. merula*) | WEU |
|  |  | *P.* cf. *vaughani* | 1 | pTUMER15 | Turdidae (*T. merula*) | WEU |
|  |  | *P.* cf. *vaughani* | 1 | pTUMER16 | Turdidae (*T. merula*) | WEU |
|  |  | *P.* cf. *vaughani* | 1 | pTURPEL04 | Turdidae (*T. pelios*) | CAF |
|  |  | *P.* cf. *vaughani* |  | pSTURUNI01 | Sturnidae | WEU |
|  |  | *P.* cf. *vaughani* |  | pSTURUNI02 | Sturnidae | WEU |
|  | II | *P.* sp. | 1 | pAFTRU4 | Turdidae (*T. libonyanus, T. pelios*) | CAF |
|  |  | *P.* sp. |  | pCOREG01 | Sturnidae | EAF |
|  | III | *P.* sp. | 1 | pTURPEL05 | Turdidae (*T. pelios*) | NAF |
|  | IV | *P. unalis* | 1 | pTFUS06 | Turdidae (*T. fuscater*) | SAM |
|  |  | *P. unalis* | 1 | pTURUF03 | Turdidae (*T. rufiventris, T. leucomelas*) | SAM |
|  |  | *P. unalis* | 1 | pTURUF04 | Turdidae (*T. rufiventris*) | SAM |
|  |  | *P. unalis* | 1 | pTUMIG03 | Turdidae (*T. falcklandii, T. amaurochalinus*, *T. albicollis, T. nigriceps, T. flavipes,* etc.), etc. | SAM, CAM, NAM |
|  |  | *P.* cf. *unalis* | 1 | pHYLMUS01 | Turdidae (*H. mustelina*) | NAM |
|  |  | *P.* cf. *unalis* | 1 | pTUMIG23 | Turdidae (*T. migratorius*) | NAM |
|  |  | *P.* cf. *unalis* | 1 | pTURALB02 | Turdidae (*T. albicollis*) | SAM |
|  |  | *P.* cf. *unalis* | 1 | pTURFAL03 | Turdidae (*T. falcklandii*) | SAM |
|  |  | *P.* cf. *unalis* | 1 | pTURFAL04 | Turdidae (*T. falcklandii*) | SAM |
|  |  | *P.* cf. *unalis* | 1 | pTURASS03 | Turdidae (*T. assimilis*) | CAM |
|  |  | *P.* cf. *unalis* |  | pCOETOR01 | Trochilidae | SAM |
|  |  | *P.* cf. *unalis* |  | pCONCIN01 | Thraupidae | SAM |
|  |  | *P.* cf. *unalis* |  | pTANICT02 | Thraupidae | CAM |
|  | V | *P.* sp. | 1 | pTURALB03 | Turdidae (*T. albicollis, T. amaurochalinus*), Dendrocolaptidae | SAM |
|  |  | *P.* sp. | 1 | pTURALB05 | Turdidae (*T. albicollis*) | SAM |
|  | VI | *P.* sp. | 1 | pTURFLA01 | Turdidae (*T. flavipes*) | SAM |
|  |  | *P.* sp. | 1 | pTURFAL06 | Turdidae (*T. flavipes*) | SAM |
|  | VII | *P.* sp. | 1 | pTULEU01 | Turdidae (*T. leucomelas, T. amaurochalinus*), Tyrannidae, Thamnophilidae | SAM |
|  |  | *P.* sp. | 1 | pTULEU04 | Turdidae (*T. leucomelas*) | SAM |
|  |  | *P.* sp. | 1 | pTULEU05 | Turdidae (*T. leucomelas*) | SAM |
|  |  | *P.* sp. | 1 | pTULEU06 | Turdidae (*T. leucomelas*), Tyrannidae | SAM |
|  |  | *P.* sp. | 1 | pTULEU07 | Turdidae (*T. leucomelas, T. amaurochalinus*) | SAM |
|  |  | *P.* sp. | 1 | pTULEU08 | Turdidae (*T. leucomelas*) | SAM |
|  |  | *P.* sp. |  | pGARUF01 | Galbulidae | SAM |
|  |  | *P.* sp. |  | pMASTR01 | Thraupidae, Bucconidae | SAM, CAM |
|  | VIII | *P.* sp. | (1) | pTHASAT06 | Thamnophilidae, Turdidae (*T. fumigatus*) | SAM |
|  |  | *P.* sp. |  | pCANLEU02 | Troglodytidae | SAM |
|  |  | *P.* sp. |  | pHYPSTR15 | Thamnophilidae | SAM |
| pTUR2 | I | *P. matutinum* | 1 | pLINN1 | Turdidae (*T. merula, T. philomelos, T. migratorius*), etc. | WEU, EEU, WAS, NAM |
|  |  | *P. matutinum* | 1 | pAFTRU5 | Turdidae (*T. merula, T. migratorius, T. unicolor*), etc. | WEU, EEU, WAS, NAF, NAM, SAS |
|  |  | *P.* cf. *matutinum* | 1 | pTUMER06 | Turdidae (*T. merula*) | WEU |
|  |  | *P.* cf. *matutinum* | 1 | pTUMER12 | Turdidae (*T. merula*) | WEU |
|  |  | *P.* cf. *matutinum* | 1 | pTUPHI08 | Turdidae (*T. philomelos*) | WEU |
|  |  | *P.* cf. *matutinum* | 1 | pTUPHI09 | Turdidae (*T. philomelos*) | WEU |
|  |  | *P.* cf. *matutinum* | | pPHACAR01 | Phalacrocoracidae | EAS |
|  | II | *P. lutzi* | 1 | pCATUST05 | Turdidae (*T. migratorius, T. falcklandii, T. fuscater, T. chiguanco, M. ralloides*), Strigidae, Paridae, etc. | NAM, SAM |
|  |  | *P. lutzi* | 1 | pTFUS05 | Turdidae (*T. fuscater*) | SAM |
|  |  | *P.* cf. *lutzi* | 1 | pTUMIG22 | Turdidae (*T. migratorius, T. plumbeus*) | NAM, CAM, CAR |
|  |  | *P.* cf. *lutzi* |  | pDIGLAF01 | Thraupidae, etc. | SAM |
|  |  | *P.* cf. *lutzi* |  | pDIGLAF02 | Thraupidae | SAM |
|  |  | *P.* cf. *lutzi* |  | pDIGCYA08 | Thraupidae | SAM |
|  | III | *P.* sp. | 1 | pSPMAG06 | Turdidae (*T. rufiventris*) | SAM |
|  |  | *P.* sp. | 1 | pTUAMA01 | Turdidae (*T. amaurochalinus, T. albicollis, T. leucomelas, T. leucops*) | SAM |
|  |  | *P.* sp. | 1 | pTULEU03 | Turdidae (*T. leucomelas*) | SAM |
|  |  | *P.* sp. | 1 | pTURALB01 | Turdidae (*T. rufiventris, T. flavipes, T. albicollis, T. nigriceps*) | SAM |
|  |  | *P.* sp. | 1 | pTURAMA01 | Turdidae (*T. amaurochalinus*) | SAM |
|  | IV | *P.* sp. | 1 | pTUROLI01 | Turdidae (*T. olivaceofuscus*) | CAF |
|  |  | *P.* sp. | 1 | pTUROLI11 | Turdidae (*T. olivaceofuscus*) | CAF |
|  | V | *P.* sp. | 1 | pTURPEL03 | Turdidae (*T. pelios*) | CAF |
|  |  | *P.* sp. |  | pLUME2 | Muscicapidae | WEU |
|  | VI | *P.* sp. | 1 | pTURPAL01 | Turdidae (*T. pallidus*) | EAS |
|  | VII | *P.* sp. | 1 | pTUROLI02 | Turdidae (*T. olivaceofuscus*) | CAF |
|  | VIII | *P.* sp. | 1 | pAFTRU09 | Turdidae (*T. pelios*) | CAF |
| pTUR3 | I | *P.* sp. | 1 | pCATUST06 | Turdidae (*C. ustulatus, M. ralloides, T. leucops, T. migratorius, T. serranus, C. aurantiirostris*), Grallariidae | SAM, CAM, NAM |
|  |  | *P.* sp. | 1 | pCATUST21 | Turdidae (*C. ustulatus*) | SAM |
|  |  | *P.* sp. | 1 | pMYARAL01 | Turdidae (*M. ralloides*) | SAM |
|  |  | *P.* sp. |  | pZOLEU01 | Emberizidae | NAM |
| pTUR4 | I | *P. homopolare* | (1) | pBAEBIC02 | Parulidae, Trochilidae, Emberizidae, Turdidae (*T. leucops, T. nigriceps, M. ralloides*), Thraupidae, Troglodytidae | SAM, NAM, CAM |
|  |  | *P. homopolare* |  | pZOCAP12 | Emberizidae | NAM |
|  |  | *P.* cf. *homopolare* | 1 | pLAIRI01 | Parulidae, Thraupidae, Turdidae (*S. mexicana, C. ustulatus*), Odontophoridae | NAM, PAC |
|  |  | *P.* cf. *homopolare* | 1 | pCURCUR01 | Turdidae (*T. falcklandii*), Icteridae | SAM |
|  |  |  | Sum=56 |  |  |  |

Geographic and host distribution of *Plasmodium* lineages contained in the haplotype networks (Figures 1 to 3). The main host families (and host species in case of Turdidae) and geographic region of origin according to the United Nations geo-scheme (with slight modifications) are indicated for each lineage. Underlined codes indicate lineages, which were analyzed morphologically in previous studies. Lineages, which are specific to or common in thrushes, are marked. The abbreviations of the regions are as following: CAF (Central Africa), CAM (Central America), CAR (Caribbean), EAF (Eastern Africa), EAS (Eastern Asia), EEU (Eastern Europe), NAF (Northern Africa), NAM (North America), SAM (South America), SAS (Southern Asia), WAS (Western Asia), WEU (Western Europe), and PAC (Pacific).
