# Supplementary material for: Geographic and host distribution of haemosporidian parasite lineages from birds of the family Turdidae
Source: Malar J. 2020 Sep 15;19:335. doi: 10.1186/s12936-020-03408-0 (PMC7491118; doi:10.1186/s12936-020-03408-0)
Supplement: Supplementary file 6 — Additional file 6. Additional Haemoproteus lineages found in Turdidae birds. [file 12936_2020_3408_MOESM6_ESM.docx]

**Additional file 6: Additional *Haemoproteus* lineages found in Turdidae birds**

| **Group** | **Species** | **Turdidae-specific** | **Lineage** | **Main host groups (families)** | **Records from Turdidae (Species)** | **Region** |
| --- | --- | --- | --- | --- | --- | --- |
| 1 | *H.* sp. | 1 | hAFR130 | Turdidae, Pycnonotidae | *Zoothera gurneyi* (1) | EAF |
| 2 | *H.* sp. | 1 | hCATGUT01 | Turdidae | *C. guttatus* (1) | NAM |
| 3 | *H.* sp. | 1 | hCATUST07 | Turdidae | *C. ustulatus* (1) | NAM |
| 4 | *H.* sp. |  | hCATUST10 | Fringillidae | *C. ustulatus* (1) | NAM |
| 5a | *H.* sp. | 1 | hCATUST15 | Turdidae | *C. ustulatus* (1) | NAM |
| 5b | *H.* sp. | 1 | hCATUST17 | Turdidae | *C. ustulatus* (1) | NAM |
| 6a | *H.* sp. | 1 | hCATUST16 | Turdidae, Parulidae, Vireonidae | *C. ustulatus* (1) | NAM |
| 6b | *H.* sp. | 1 | hCATUST18 | Turdidae | *C. ustulatus* (1) | NAM |
| 6c | *H.* sp. |  | hVIGIL09 | Vireonidae, etc. | *T. nigriceps* (1) | SAM |
| 7 | *H.* sp. | 1 | hCATUST19 | Turdidae | *C. ustulatus* (1) | CAM |
| 8 | *H.* sp. |  | hCOLPAS04 | Columbidae, Tyrannidae, etc. | *T. assimilis* (1) | CAM |
| 9 | *H.* sp. |  | hCYCYAN01 | Fringillidae | *T. assimilis* (1) | CAM |
| 10a | *H.* sp. |  | hDUNNO01 | Fringillidae, Passeridae | *Ixoreus naevius* (1) | NAM |
| 10b | *H.* sp. |  | hSPIARB01 | Fringillidae, etc. | *C. ustulatus* (2) | NAM |
| 11 | *H. brachiatus* |  | hLK03 | Falconidae | *T. merula* (1) | WEU |
| 12 | *H.* sp. |  | hSIAMEX01 | Fringillidae, Icteridae, etc. | *Sialia mexicana* (1)*, S. sialis* (1) | NAM |
| 13a | *H. witti* | 1 | hTROAED20 | Trochilidae, Tyrannidae, Turdidae, Parulidae, Funariidae, Certhiidae, Fringillidae, etc. | *M. ralloides* (6)*, T. nigriceps* (1)*, T. serranus* (1) | SAM |
| 13b | *H.* cf. *witti* | 1 | hTFUS16 | Turdidae | *T. fuscater* (1) | SAM |
| 14 | *H.* sp. | 1 | hTURUF02 | Turdidae | *T. rufiventris* (1) | SAM |
| 15 | *H. erythrogravidus* |  | hZOCAP14 | Fringillidae | *C. fuscater* (1) | SAM |
|  |  | Sum=11 | Sum=20 |  |  |  |

*Haemoproteus* lineages rarely found in Turdidae birds. The main host families are indicated for each lineage. The Turdidae hosts in which these lineages were found and the geographic region (United Nations geo-scheme with slight modifications) of origin are indicated as well. Underlined codes indicate lineages, which were analyzed morphologically in previous studies. Lineages, which are specific to or common in thrushes, are marked. The data on the occurrence of the lineages in bird hosts originates from MalAvi database (http://130.235.244.92/Malavi/). The abbreviations of the regions are as following: CAM (Central America), EAF (Eastern Africa), NAM (North America), SAM (South America), and WEU (Western Europe).
