# Supplementary material for: Geographic and host distribution of haemosporidian parasite lineages from birds of the family Turdidae
Source: Malar J. 2020 Sep 15;19:335. doi: 10.1186/s12936-020-03408-0 (PMC7491118; doi:10.1186/s12936-020-03408-0)

**Phylogenetic tree of avian *Haemoproteus* lineages from birds of the Turdidae**

Maximum Likelihood tree calculated based on an alignment of *CytB* sequences (474 bp) from avian haemosporidian parasites.

Maximum Likelihood boostrap values and Bayesian posterior probabilities are indicated at most nodes.

Lineages in bold type were isolated from Turdidae birds, lineages in regular type from other bird families.

Labelled clades comprise lineages contained in the DNA hyplotype networks.

The scale bar indicates the expected mean number of substitutions per site according to the model of sequence evolution applied.

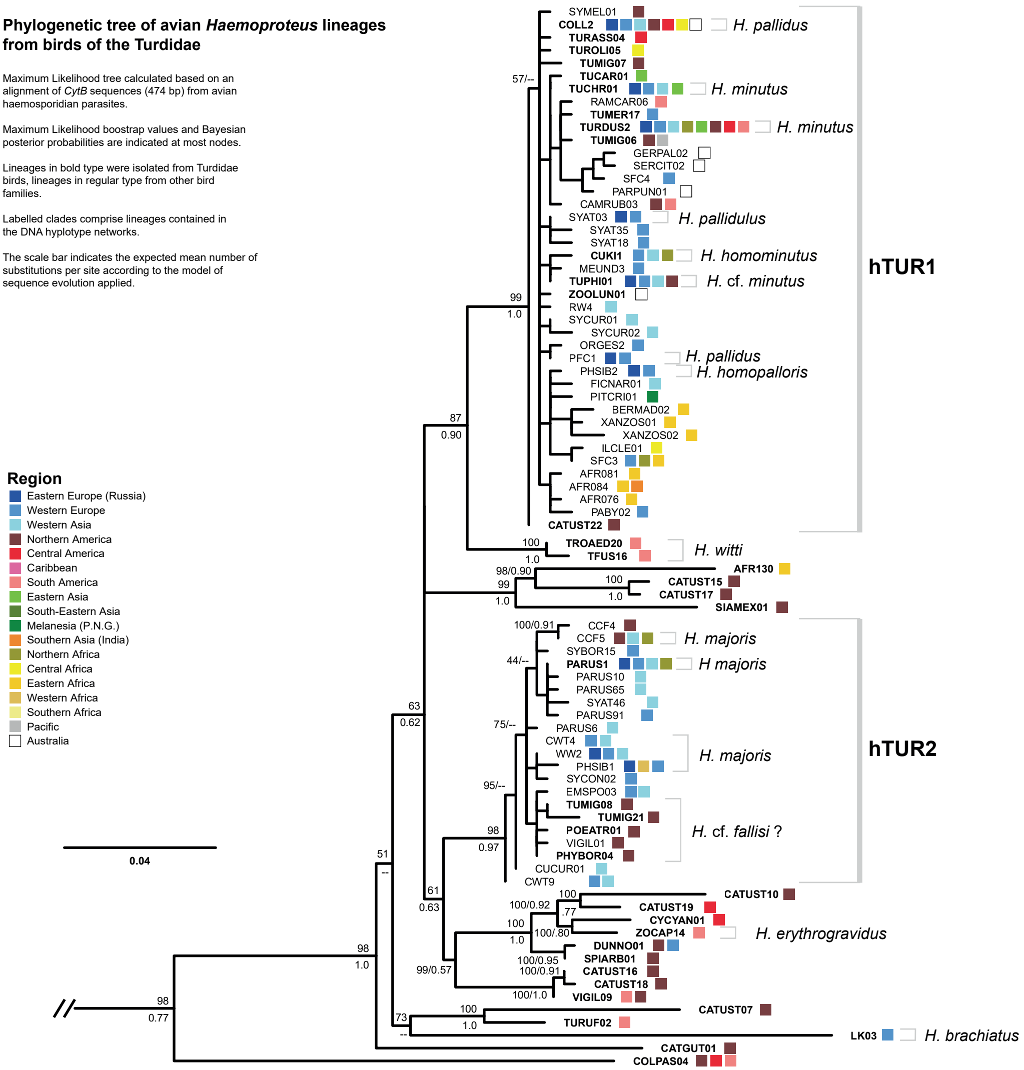

Supplement: Supplementary file 7 — Additional file 7. Maximum likelihood tree of Haemoproteus CytB lineages (474 bp) included in the present study. The total geographic distribution of lineages according to the United Geo-scheme is based on data from the MalAvi data base. [file 12936_2020_3408_MOESM7_ESM.pdf]
