# Supplementary material for: Geographic and host distribution of haemosporidian parasite lineages from birds of the family Turdidae
Source: Malar J. 2020 Sep 15;19:335. doi: 10.1186/s12936-020-03408-0 (PMC7491118; doi:10.1186/s12936-020-03408-0)
Supplement: Supplementary file 10 — Additional file 10. Additional Leucocytozoon lineages found in Turdidae birds. [file 12936_2020_3408_MOESM10_ESM.docx]

**Additional file 10: Additional *Leucocytozoon* lineages found in Turdidae birds**

| **Group** | **Species** | **Turdidae-specific** | **Lineage** | **Main host groups (families)** | **Records from Turdidae (Species)** | **Region** |
| --- | --- | --- | --- | --- | --- | --- |
| 1a | *L.* sp. |  | ASOT06 | Strigiformes | *T. merula* (3) | WEU |
| 1b | *L.* sp. | 1 | TUMER20 | Turdidae | *T. merula* (1) | WEU |
| 2a | *L.* sp. |  | CATUST14 | Parulidae, etc. | *C. ustulatus* (1)*, M. occidentalis* (3) | NAM |
| 2b | *L.* sp. | 1 | MYAUNI01 | Turdidae | *M. unicolor* (2) | CAM |
| 2c | *L.* sp. | 1 | MYAUNI03 | Turdidae | *M. unicolor* (1) | CAM |
| 2d | *L.* sp. |  | COLBF21 | Fringillidae, Parulidae, Paridae, Vireonidae, etc. | *H. mustelina* (1)*, S. mexicana* (1) | NAM |
| 3a | *L.* sp. |  | DUMCAR01 | Fringillidae, Parulidae, Mimidae | *C. fuscescens* (2) | NAM |
| 3b | *L.* sp. |  | TUMIG12 | Fringillidae, Parulidae | *T. migratorius* (1) | NAM |
| 3c | *L.* sp. | 1 | TROAED02 | Certhiidae, Fringillidae, Tyrannidae, Parulidae, Turdidae | *M. ralloides* (1) | SAM |
| 3d | *L.* sp. | 1 | MYARAL02 | Turdidae | *M. ralloides* (1) | SAM |
| 4a | *L.* sp. | 1 | TFUS14 | Turdidae | *T. fuscater* (1) | SAM |
| 4b | *L.* sp. | 1 | TUMER18 | Turdidae | *T. merula* (1) | WEU |
| 5 | *L.* sp. | 1 | AFR187 | Turdidae | *Geokichla gurneyi* (1) | EAF |
| 6 | *L.* sp. |  | CAP3 | Phasianidae | *T. pilaris* (1) | WEU |
| 7 | *L.* sp. | 1 | CATUST34 | Turdidae | *C. ustulatus* (1) | NAM |
| 8 | *L.* sp. |  | METYR01 | Fringillidae, Trochilidae, Tyrannidae, Turdidae | *T. fuscater* (1) | SAM |
| 9 | *L.* sp. | 1 | TUMER03 | Turdidae | *T. merula* (2) | WEU, WAS |
| 10 | *L.* sp. | 1 | TUMER09 | Turdidae | *T. merula* (1) | WEU |
| 11 | *L.* sp. | 1 | TUMER10 | Turdidae | *T. merula* (1) | WEU |
| 12 | *L.* sp. | 1 | TFUS15 | Turdidae | *T. fuscater* (1) | SAM |
| 13 | *L.* sp. | 1 | TUMIG13 | Turdidae | *T. migratorius* (1) | NAM |
| 14 | *L.* sp. | 1 | TUMIG14 | Turdidae | *T. migratorius* (1) | NAM |
| 15 | *L.* sp. | 1 | TURALB04 | Turdidae | *T. albicollis* (1) | SAM |
| 16 | *L.* sp. | 1 | TURMIG09 | Turdidae | *T. migratorius* (1) | NAM |
| 17 | *L.* sp. | 1 | TUROLI06 | Pycnonotidae, Turdidae | *T. olivaceofuscus* (1) | CAF |
|  |  | Sum=18 | Sum= 25 |  |  |  |

*Leucocytozoon* lineages rarely found in Turdidae birds. The main host families are indicated for each lineage. The Turdidae hosts in which these lineages were found and the geographic region (United Nations geo-scheme with slight modifications) of origin are indicated as well. Lineages, which are specific to or common in thrushes, are marked. The data on the occurrence of the lineages in bird hosts originates from MalAvi database (http://130.235.244.92/Malavi/). The abbreviations of the regions are as following: CAF (Central Africa), CAM (Central America), EAF (Eastern Africa), NAM (North America), SAM (South America), and WEU (Western Europe).
